# Structural and non-structural proteins in SARS-CoV-2: potential aspects to COVID-19 treatment or prevention of progression of related diseases

**DOI:** 10.1186/s12964-023-01104-5

**Published:** 2023-05-15

**Authors:** Sareh Kakavandi, Iman Zare, Maryam VaezJalali, Masoud Dadashi, Maryam Azarian, Abdullatif Akbari, Marzieh Ramezani Farani, Hamidreza Zalpoor, Bahareh Hajikhani

**Affiliations:** 1 Department of Bacteriology and Virology, School of Medicine, Shiraz University of Medical Sciences, Shiraz, Iran; 2 Research and Development Department, Sina Medical Biochemistry Technologies Co. Ltd., Shiraz, 7178795844 Iran; 3 Department of Microbiology, School of Medicine, Shahid Beheshti University of Medical Sciences, Tehran, Iran; 4 Department of Microbiology, School of Medicine, Alborz University of Medical Sciences, Karaj, Iran; 5 Non-Communicable Diseases Research Center, Alborz University of Medical Sciences, Karaj, Iran; 6 Department of Radiology, Charité - Universitätsmedizin Berlin, 10117 Berlin, Germany; 7 Shiraz Neuroscience Research Center, Shiraz University of Medical Sciences, Shiraz, Iran; 8 Network of Immunity in Infection, Malignancy and Autoimmunity (NIIMA), Universal Scientific Education and Research Network (USERN), Tehran, Iran; 9 Department of Biological Sciences and Bioengineering, Nano Bio High-Tech Materials Research Center, Inha University, Incheon, 22212 Republic of Korea

**Keywords:** SARS-CoV-2, COVID-19, Structural proteins, Non-structural proteins, Vaccine

## Abstract

Coronavirus disease 2019 (COVID-19) is caused by a new member of the Coronaviridae family known as severe acute respiratory syndrome coronavirus 2 (SARS-CoV-2). There are structural and non-structural proteins (NSPs) in the genome of this virus. S, M, H, and E proteins are structural proteins, and NSPs include accessory and replicase proteins. The structural and NSP components of SARS-CoV-2 play an important role in its infectivity, and some of them may be important in the pathogenesis of chronic diseases, including cancer, coagulation disorders, neurodegenerative disorders, and cardiovascular diseases. The SARS-CoV-2 proteins interact with targets such as angiotensin-converting enzyme 2 (ACE2) receptor. In addition, SARS-CoV-2 can stimulate pathological intracellular signaling pathways by triggering transcription factor hypoxia-inducible factor-1 (HIF-1), neuropilin-1 (NRP-1), CD147, and Eph receptors, which play important roles in the progression of neurodegenerative diseases like Alzheimer's disease, epilepsy, and multiple sclerosis, and multiple cancers such as glioblastoma, lung malignancies, and leukemias. Several compounds such as polyphenols, doxazosin, baricitinib, and ruxolitinib could inhibit these interactions. It has been demonstrated that the SARS-CoV-2 spike protein has a stronger affinity for human ACE2 than the spike protein of SARS-CoV, leading the current study to hypothesize that the newly produced variant Omicron receptor-binding domain (RBD) binds to human ACE2 more strongly than the primary strain. SARS and Middle East respiratory syndrome (MERS) viruses against structural and NSPs have become resistant to previous vaccines. Therefore, the review of recent studies and the performance of current vaccines and their effects on COVID-19 and related diseases has become a vital need to deal with the current conditions. This review examines the potential role of these SARS-CoV-2 proteins in the initiation of chronic diseases, and it is anticipated that these proteins could serve as components of an effective vaccine or treatment for COVID-19 and related diseases.

Video Abstract

**Supplementary Information:**

The online version contains supplementary material available at 10.1186/s12964-023-01104-5.

## Introduction

**Fig. 1:**
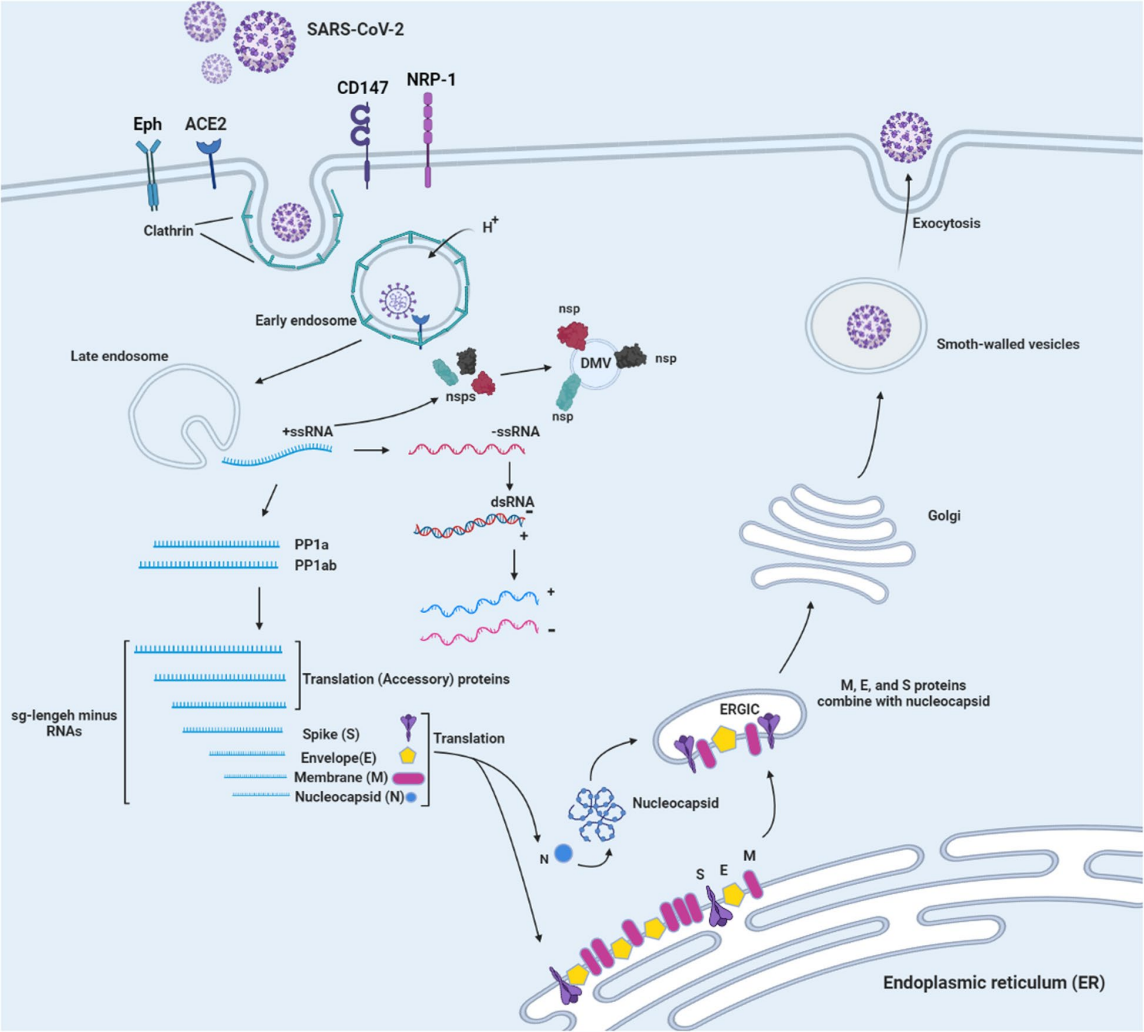
SARS-CoV-2 entry receptors (ACE2, Eph, NRP-1, and CD147). Life cycle and role of proteins in replication of SARS-CoV-2

In late January 2019, Wuhan, China, experienced the first outbreak of the novel coronavirus disease, COVID-19, which then spread rapidly worldwide [1, 2]. The virus that causes the highly contagious disease COVID-19 is called SARS-CoV-2 [3, 4]. This virus has a single-stranded RNA genome with a positive sense. Microscopic studies show that this virus has crown-shaped ridges on its surface, which shows that it belongs to the family of coronaviruses [5–7]. Typically, the virus is structurally arranged in a 50-cap structure, followed by the leader sequence, the untranslated region (UTR), and the sequences encoding the replication polyproteins and accessory and structural proteins, and finally, the UTR regions and poly-A tail is coded [8, 9]. On the other hand, the replicase gene called open reading frame 1ab (ORF1ab) occupies two-thirds of the virus genome, located downstream to the 50′ ends. This region encodes NSPs, called pp1a and pp1ab proteins, respectively [10, 11]. In addition, nonstructural pp1a proteins include NSP1 to NSP11 and nonstructural pp1a proteins include NSP12 to NSP16 [11, 12]. Finally, the remaining region before the 30′ end encodes the structural proteins including S, M, N, and E proteins [12, 13]. Also, structural proteins encode nine accessory proteins, which are encoded by ORF3a, ORF3d, ORF6, ORF7a, ORF7b, ORF8, ORF9b, and ORF10 genes [14]. The pathogenicity of this virus is the beginning of the entry of the virus into the host cell by binding to the ACE2 receptor through its S protein [14]. There are two ways for this virus to reach the host cell: 1) cutting in S1 and S2 sites, which is done by surface membrane serine 2 proteases [15]. 2) endolysosomal cathepsin L, which causes the cell membrane of the virus to fuse at the cell surface and endosomes [16]. Once the RNA genome is released into the cytoplasm of the host cell, replication begins in the endoplasmic reticulum (ER)-derived double-membrane vesicles (DMVs) [17], and these DMVs combine to form a complex network of membranes [18]. Then the positive strand genome acts as a template for negative-sense RNA and RNA(sg) [19]. The translation of RNA leads to the formation of structural and peripheral proteins whose function is to assemble the virion in the middle part of the Golgi [20]. Finally, the positive genome is synthesized into new virions and the host cell is infected with this virus [12] (Fig. 1). It can be concluded that these studies facilitate our understanding of how this infection occurs and lead to the development of more efficient antiviral and therapeutic strategies.

For example, it has been reported that the Omicron variant can prevent neutralization by sera obtained from individuals who have received only one or two doses of the vaccine, especially when antibody titers are low [21]. Three doses of the spike-based vaccine may provide only partial protection against infection with present strains [22, 23]. In countries with high rates of vaccination or natural immunity, Omicron evasion of the immune system may have contributed to extraordinarily high rates of transmission [24]. Also, due to the continuous mutations new variants such as Delta variants emerge with higher or different manifestations of COVID-19 appear [25] (Table 1).

**Table 1:** Mutations and evolution of the SARS-CoV-2 variants

Types of variants	Mutations	Targets	Refs
Alpha (B.1.1.7 lineage)	• 17 mutations	• The viral genome	[26–28]
	• 8 mutations (especially N501Y)	• Increased spike protein binding to ACE 2 receptors • Making the viral attachment stronger	
Beta (B.1.351 lineage)	• 9 mutations (D614G, D80A, D215G, R246I, K417N, E484K, N501Y, L18F and A701V)	• S protein	[27, 29, 30]
	• 3 mutations (K417N, E484K, and N501Y)	• Located in the RBD and increase the ACE receptors' binding affinity	
Gamma (P.1 lineage)	• 10 mutations (L18F, T20N, P26S, D138Y, R190S, H655Y, T1027I V1176, K417T, E484K, and N501Y)	• Spike protein	[27, 31, 32]
	• 3 mutations (L18F, K417N, E484K)	• Similar to the B.1.351 variant and located in the RBD	
Delta (B.1.617.2 lineage)	• 10 mutations (T19R, R158G, (G142D), L452R, T478K, D614G, P681R, D950N,156del, 157del)	• Effect on spike protein	[27, 33]
Omicron (B.1.1.529 lineage)	• 30 mutations (T91, G204R, P13L, E31del, R32del, R203K,S33del, D3G, A63T, Q19E, N211/L212I, G142D, T95I, V70, H69, A67V, Y145del, Y144del, Y143del, G496S, Q493R, E484A, T478K, S477N, Q498R, G446S, N440K, S375F, S373P, S371L, G339D, N501Y,Y505H,K417N, D796Y, L981F, Q954H, N969K	• Envelope • Nucleocapsid protein • Matrix • Domain at the spike protein's N-terminus • The RBD of the spike • Fusion peptide of the spike • Spike protein and NSPs	[27, 34, 35]

### Spike protein

The 200 nm long spike (S) protein located on the surface of the viral membrane between amino acids 1160 and 1450 contributes to the fusion of the viral membrane with the host cell membrane [36, 37]. This protein is a multifunctional molecular machine that is released from the virus in the form of a crown [38]. A single-pass anchor, a short intracellular tail, and S1 and S2 subunits constitute the three structural components of the S protein [39, 40]. S1, which binds to the host ACE2 receptor, and S2, which mediates the fusion of the viral cell membrane with the host [41, 42]. Two envelope glycoproteins of SARS-CoV-2, the S and membrane (M) proteins, are essential for virus pathophysiology [43, 44]. Several host cell proteases, including furin, trypsin, cathepsin, and TMPRSS2 (transmembrane protease serine subclass 2), degrade this protein [45, 46]. The presence of proteases in the target cell determines the ways to enter the virus through the host cell membrane, plasma, or endocytosis [47, 48]. In other words, the virus enters the cell by binding to the receptors of the host cell if this protein is present, or the junction of the virus to the host cell membrane facilitates the entry [49, 50]. The S2 subunit fuses the virus to the host cell membrane and allows the virus to enter the host cell, while the S1 subunit binds the virus to the host cell [51]. This protein contains the most important antigens that are responsible for neutralization by antibodies and are the target of cytotoxic cells [52, 53]. The main role of the S protein is to increase the contact of the cell membrane with the viral membrane, which can be a target for treatment with antibodies or chemical compounds or a target for vaccination [54, 55] (Table 2).

**Table 2:** The summary of drugs and vaccines with their targets, structures, and advantages

Drugs/vaccines & companies	Targets	Structures	Advantages	Refs
**Inactivated and protein subunit vaccines**				
Sinopharm CanSinoBIO Sinovac AstraZeneca Sputnik V	• Spike protein	• A virus that has been chemically inactivated after being grown in a culture	• 86% effectivity of Sinopharm	[56, 57]
**Nucleic acid vaccines**				
**DNA vaccines**				
LineaRx Takis Biotech ZyCoV-D vaccine	• Enhance the induction of T cells • Different forms of the SARS-CoV-2 S protein are encoded by this gene • The response was mediated by type I helper T cells (Th1) rather than type II helper T cells (Th2)	• Viral genetic sequence-based and S protein genetic sequence-based)	• The safety and efficacy • Humoral and cellular immunity	[58–60]
**Vector vaccines**				
Houston-based Greffex Inc Adenovirus Type 5 Vector ChAdOx1 nCoV-19 Adeno-based (rAd26-S + rAd5-S) Ad26COVS1 Johnson & Johnson AstraZeneca	• Engineered viruses incapable of replication	• An adenovirus vector or other vector for the construction of SARS-CoV-2	• Elicits the innate immune responses that are necessary for adaptive immune responses • Effective in avoiding hospitalization and death caused by COVID-19	[56, 58, 59]
**mRNA vaccines**				
Moderna Pfizer BioNTech German biopharmaceutical ZY Therapeutics CanSino CureVac AG Stermirna Therapeutics Guanhao Biotech Therapeutics BDGENE	• SARS-CoV-2 S protein and RBD domain	• Lipid nanoparticles	• 90% effectiveness against the clinical disease caused by SARS-CoV-2, with very few side effects • The rapidity of vaccine production • Ability to produce responses to TH1 and TH2 • Vaccines for children and common SARS-CoV-2 variants that are approved	[56, 58, 61]
**Subunit vaccines**				
Novavax Johnson & Johnson Chongqing Zhifei Sanofi Pasteur/GSK	• Viral proteins are injected into the host	• Recombinant SARS-CoV-2 in its entirety glycoprotein nanoparticle vaccine adjuvanted	• The best alternative is vaccines with adjuvanted subunits	[58, 59, 62]
**Virus-like particles vaccines**				
Medicago Inc	• Proteins from the viral capsid • Induced immunity against SARS-CoV-2	• VLP is derived from plants and adjuvanted with Dynavax or GSK adjuvants • VLP vaccines are recombinant genetically modified viruses that are generally thought to be safe because they fail to replicate • Whole S protein	• Immunogenicity and safety advantages	[58, 63]
**Drugs**				
Arbidol (umifenovir)	• Targets ACE2 protein and S protein interaction causes the viral envelope's membrane fusion to be prevented • Reduce the SARS virus's envelope's fusion	• Advantages against SARS-CoV-2		[64, 65]
Camostat	• TMPRSS2 inhibitor of the host serine protease	• Advantages against SARS-CoV-2		[64, 65]
Hydroxychloroquine	• Acts on the spike glycoprotein-ganglioside receptor and its interaction with the ACE2 receptor • Reduced endosome acidification and attenuation of host receptor glycosylation, proteolytic processing, cytokine production, lysosomal activity, autophagy, and endocytic pathways • Enhances intracellular pH, eventually affects cathepsins, inhibits antigen-presenting cells (APCs), autophagosomal functions, MAP kinase, and autophagosomal functions • Structural damage to SARS-CoV-2's spike proteins	• Anti-inflammatory effect on IL-17, IL-122, and IL-6 cytokines		[64, 65]
Remdesivir	• Use RdRp to stop translation and replication processes • Inhibits NSP12 in other coronaviruses	• As evidenced by the low polymerase activity in host cells during the Ebola virus outbreak • Undergoing in patients with mild, moderate, and severe SARS-CoV-2		[64, 65]

### Specific receptors of spike protein

#### ACE2

**Fig. 2:**
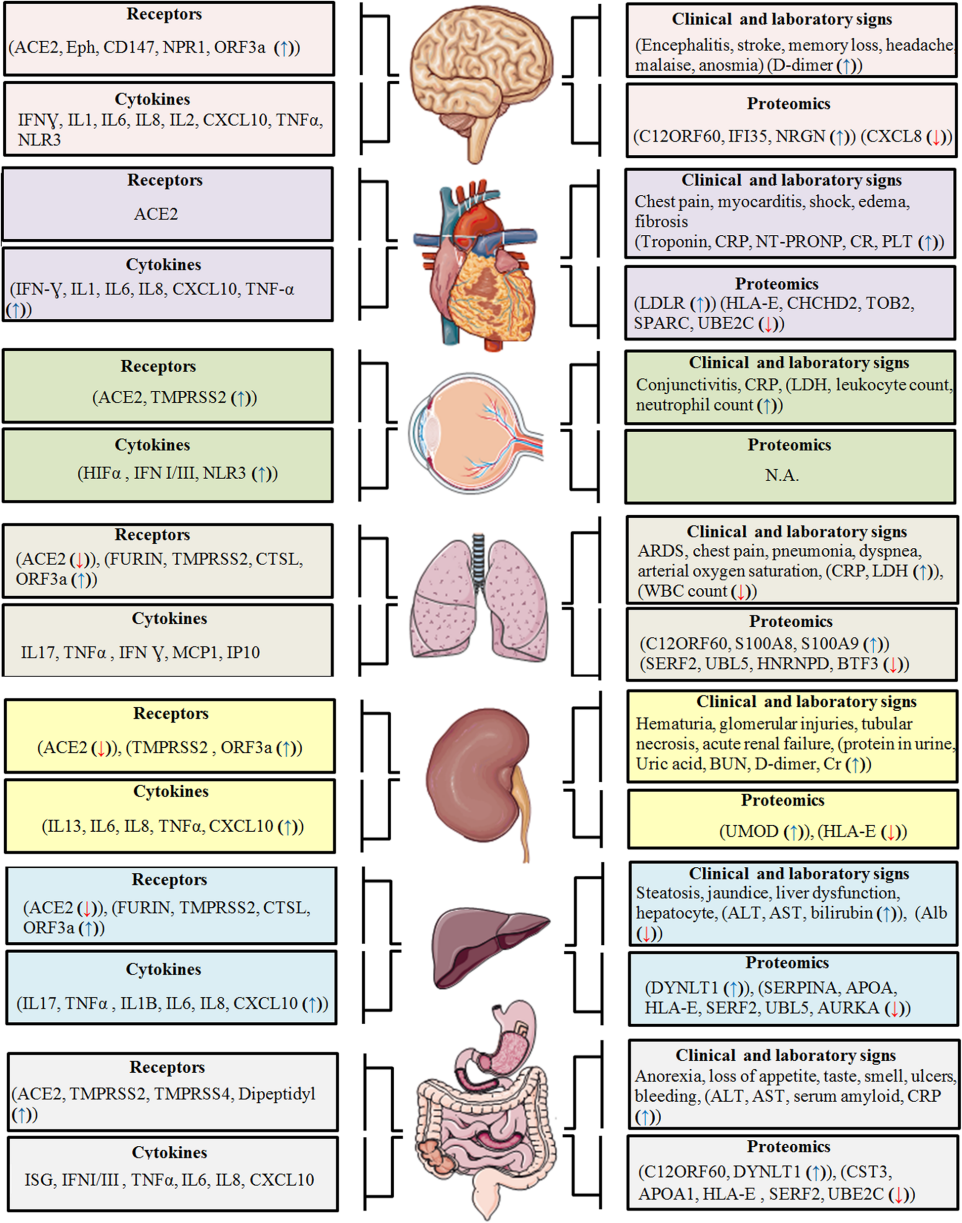
Schematic illustration of proteomics studies, clinical and laboratory signs of people infected by SARS-CoV-2, SARS-CoV-2 receptors, and cytokines expression traits in different organs infected by SARS-CoV-2. The schematic illustrations of organs are obtained from Servier Medical ART: SMART [79–94]

**Fig. 3:**
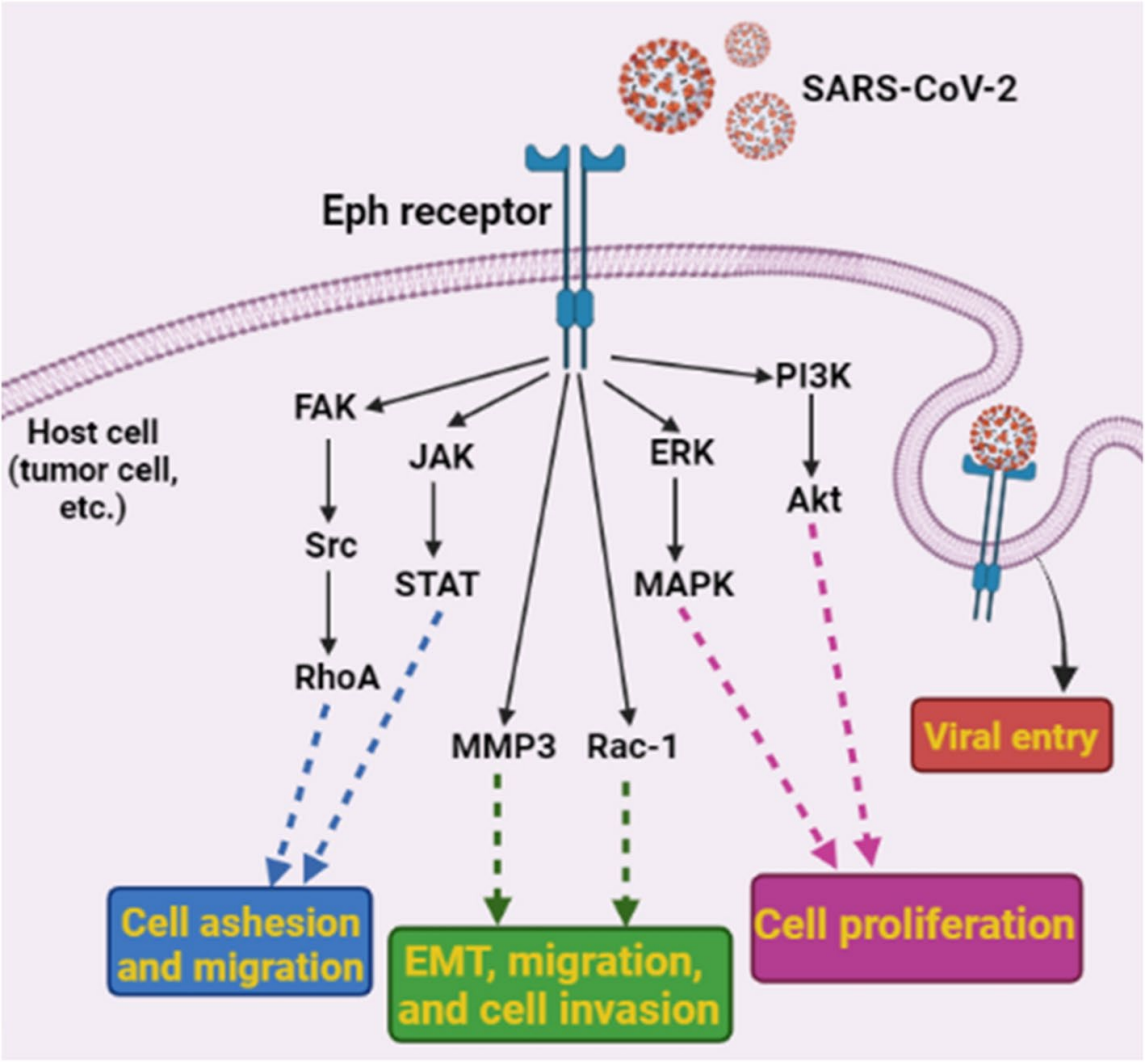
SARS-CoV-2 may have a role in cancer or other diseases by using Eph receptors as entrance receptors and activating Eph receptor downstream signaling in the host cell (especially in malignant cells). Reprinted with permission from ref. [74]. Copyright 2022, Springer Nature

**Fig. 4:**
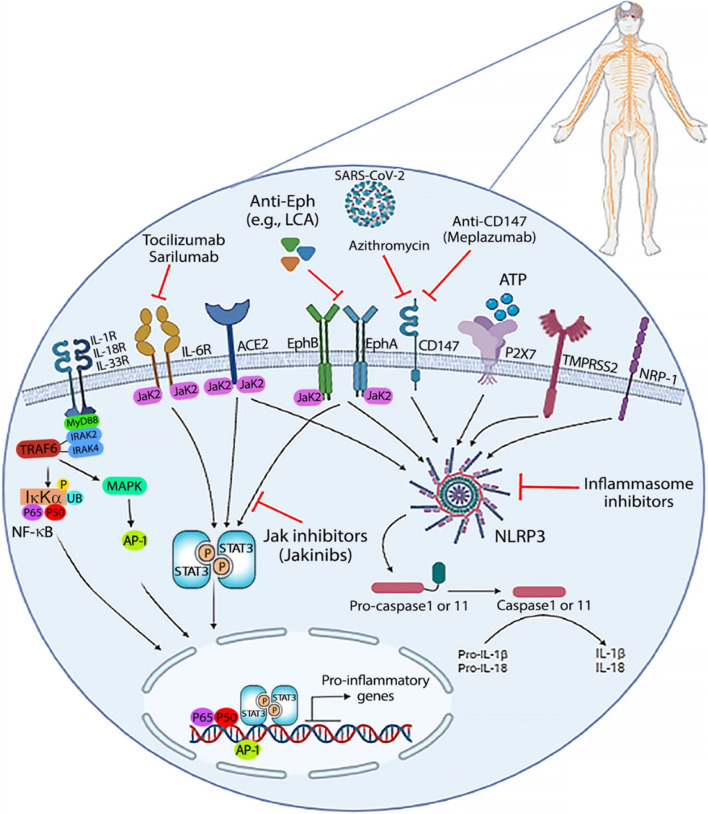
The transmembrane protease serine subclass 2 (TMPRSS2), neuropilin-1 (NRP-1), ACE2, Eph receptors, ephrin ligands, P2X7, and CD147 are expressed on cells of the CNS as SARS-CoV-2 spike protein entrance receptors. JAK inhibitors can target ACE2, EphA/B receptors, and IL-6 receptors-activated signal transduction (JAKinibs). Also, the NLRP3 inflammasome can release IL-1 and IL-18 in response to ACE2, EphA/B receptors, TMPRSS2, NRP-1, P2X7, and CD147. These molecules may be targeted by IL-1 and IL-18 monoclonal antibodies, antagonists, and inflammasome inhibitors. Reprinted with permission from ref. [73]. Copyright 2022, Springer Nature

One of the important components of COVID-19 is ACE2, which acts as a specific receptor for virus entry [66]. Multiple cell lines in the CNS express ACE2, including oligodendrocytes, microglia, astrocytes, and neurons (Fig. 2). ACE2 has shown a protective role in chronic diseases such as hypertension, acute respiratory distress syndrome, and cardiovascular diseases in the prognosis of COVID-19 [67–69] (Fig. 2). Hypertension can be significantly decreased by ACE inhibitors, which reduce the inward remodeling of arteries [70, 71]. The human brain, as well as other organs and tissues, express a unique SARS-CoV-2 receptor known as ACE2 [72]. It may help the SARS-CoV-2 virus enter host cells in the CNS in addition to ACE2, ephrin receptor, neuropilin-1 (NRP-1), and CD147. It may also trigger intracellular signaling pathways linked to pathological problems of the CNS and malignancies such as glioblastomas [73] and stimulate intracellular signaling pathways associated with CNS diseases and malignancies such as glioblastoma [73, 74] (Figs. 3 and 4). A receptor-binding site on the spike protein in the envelope of SARS-CoV-2 directly contacts the extracellular domain of ACE2 [75]. The spike protein of SARS-CoV-2 has been shown to have a stronger affinity for human ACE2 than the spike protein of SARS-CoV. For example, the newly generated variant Omicron RBD binds more strongly to human ACE2 than the main strain [76–78] (Table 1). A graphical summary of proteomics studies, clinical and laboratory signs of people infected by SARS-CoV-2, SARS-CoV-2 receptors, and cytokines expression traits in different organs infected by SARS-CoV-2 (brain, heart, eye, lung, kidney, liver, and gastrointestinal organs) [79–93] (Fig. 2).

#### Ephrin/Eph receptors

The largest human receptor tyrosine kinase (RTK) family is known as the erythropoietin-producing hepatocyte (Eph) receptors [95, 96]. The ephrin-Eph-RTK pathway controls many cellular functions including cell adhesion, proliferation, differentiation, and migration. Ephrin binds to Eph receptors as a ligand to activate it [97, 98]. Ligand-binding regions are found in the extracellular domain of Eph receptors, while regulatory domains and protein kinase regions are found in the intracellular domain [73]. Tyrosine residues on receptors become phosphorylated upon ligand activation and serve as sites where intracellular signaling proteins (or adapters) recruit and activate [73]. Eph and ephrin proteins can support viral replication, persistence, and vector-mediated viral transmission [99]. Multiple samples for viral infections can disrupt neuronal function, including the potential receptor for SARS-CoV-2 entry into human brain cells and the spike protein that acts as a stimulator of Eph receptor downstream signaling in COVID-19-related neurological diseases and cancers (Fig. 3) [73, 74, 100, 101]. These cells may serve as potential hosts for entry of SARS-CoV-2 or potentially initiate upstream signaling pathways. In Eph receptors, ligand-binding domains, cysteine-rich regions, two fibronectin III repeats (FNIII), transmembrane regions, tyrosine kinase domains, and PSD95/DLG/ZO-1 subunits are present [102]. Spike protein of SARS-CoV-2 can activate Eph receptors because Ephrin-A directly stimulates SRC and RhoA and activates FYN and ERK through focal adhesion kinase (FAK) [102]. JAK2 can be activated by STAT3 (signal transducer and activator of transcription factor 3) [103]. Activation of AKT is well known to occur in pancreatic cancer cells when EphA2 is present [104]. MMP8 (matrix metalloproteinase 8) STAT3, Src, and RAC1 are stimulated by ephrin-Bs to initiate EMT (endothelial-mesenchymal transition) [74]. EMT and invasion are triggered when these substances activate RAC1, RhoA, and CDC42. Eph/ephrin has the potential to exacerbate some prevalent diseases and age-related disorders [105]. Also encourages EphA4 forward signaling to exert its synaptotoxic effects. Consequently, EphA4/ephrin-A1 can also increase levels of endothelial cells and their supporting cells, such as smooth muscle cells and pericytes, which are also regulated by Eph/ephrin, which contributes to angiogenesis, vascular permeability, and vascular remodeling [74]. Ephrin-B2, for instance, may be expressed as a result of vascular endothelial growth factor (VEGF), because it is necessary for the endocytosis of the VEGF receptor and angiogenic signaling. Self-renewal and glioblastoma stem cell differentiation is inhibited by EphA2, an Eph receptor that is frequently overexpressed in cancers. In lung cancers, EphA2 overexpression also causes abnormal cell growth [73, 106]. Ephs/ephrins play a role in heart health and disease, and with the shape of heart tissue [107]. Age-related diseases impair EphA2 signaling for human cardiac progenitor cell migration [108]. As EphB4 is activated by EphA1/EphA2/EphA4 binding to EphB2, it promotes the adhesion of leukocytes and monocytes to endothelial cells, leading to intimal inflammation and atherosclerotic plaque formation. Therefore, the development of drugs and substances that affect and regulate the Eph/ephrin system will help in the treatment and cure of many disorders [106]. Polyphenols, doxazosin, lithocholic acid derivatives, kinase inhibitors, peptide analogs, peptide proteins, and specific antibodies are examples of small molecules that have the potential to target Eph receptors [74, 109].

#### Neuropilin-1 (NRP-1)

One of the two neuropilin homologues, neuropilin 1 (NRP-1), plays an important role in normal and pathological conditions [73]. The two isoforms of NRP-1 are secreted and transmembrane (also known as truncated or soluble NRP-1). In contrast to the latter, which binds to multiple ligands and performs different functions, the former circulates freely in physiological fluid [73, 110]. Along with ACE2, the ephrin/Eph receptor, and CD147 may facilitate the entry of the SARS-CoV-2 virus into host cells in the CNS and stimulate intracellular signaling pathways that cause CNS diseases (*e.g.*, glioblastoma) (Fig. 4) [73]. It is currently unclear how COVID-19 causes neurological disorders such as headaches, memory loss or mental disorders, insomnia, loss of taste or smell, and sleep disturbances [111, 112]. Neurological disorders, which continued 3 to 9 months after SARS-COV-2, include dizziness and depression, loss of sense of smell, memory impairment, and cognitive disorders which have been described as having a negative impact on neurological health in patients [113, 114]. NRP-1 is essential for signaling molecules including VEGF (especially VEGF-A), integrins, semaphorins, transforming growth factor-beta (TGF-β), and plexins for function [115, 116]. Some processes that rely on it include tumorigenesis, angiogenesis, virus entry, axonal guidance in the peripheral nervous system and CNS, and immunological activity [115] (Fig. 2). According to an interesting study by Cantuti-Castelvetri et al. SARS-CoV-2 can infect cells by binding to NRP-1 via the S protein, enter neurons, and then produce NRP-1 as well as two essential components, furin and transmembrane serine protease 11A (TMPRSS11A) [117]. Since olfactory epithelial cells in COVID-19 patients have high levels of NRP-1 and VEGF-A is a ligand for NRP-1, this implies that one explanation for the patient's cognitive and neurological impairments could be the sensitivity of these brain regions to SARS-CoV-2 [118]. The extracellular b1b2 domain of NFPs is involved in the binding of VEGF-A [119]. As a co-receptor for VEGFR-1 and VEGFR-2, this receptor plays an important role in angiogenesis [120]. The interaction of VEGF-A with NRP-1 leads to the formation of the GAIP/RGS19-interacting protein (GIPC1) + Syx molecular signaling complex, which facilitates RhoA GTP binding. This association is enhanced between the scaffold protein GIPC1 and NRP-1 [121]. When RhoA is activated, the tumor suppressor protein p27^kip1^ is destroyed by this active form of the protein. Therefore, this leads to the proliferation of tumor cells. Furthermore, activation of the PI3K/AKT/NF-κB pathway is thought to control NRP-1/GIPC1-mediated angiogenesis, proliferation, and migration [118, 122]. Finally, it is hypothesized that, especially in people who had multiple infections with SARS-CoV-2, the increased expression of NRP-1 caused by COVID-19 may play an important role in long-term clinical problems related to the CNS and may accelerate the development of brain tumors at the preliminary stage [73]. Pharmacological targeting of NRP-1 in such vulnerable individuals may be a valuable and potentially effective treatment to prevent long-term neurological effects and reduce the risk of neurological diseases [118] (Fig. 2).

#### CD147

Transmembrane glycoprotein CD147 belongs to the immunoglobulin superfamily (HAb18G, sometimes called EMMPRIN) [123]. The brain, T cells, endothelial cells, and many organs and cells throughout the body contain CD147 [124, 125] (Fig. 2). Although CD147 is not directly bound to SARS-CoV-2 in other viral infections, particularly SARS-CoV-2 infection, it is involved in HIV-1 infection by interacting with virus-associated cyclophilin A [126]. CD147 receptor and TMPRSS2 protease are likely to be more involved in SARS-CoV-2 CNS infection than ACE2 [127] (Fig. 2). The cerebellum and cortex and pituitary of the mouse brain have higher mRNA levels for TMPRSS2 and CD147. CD147 activity is thought to be mediated by some signaling pathways, such as MAPK p38, ERK-1/2, PI3K, and NF-κB [73, 128, 129]. ERK and IB appear to be phosphorylated when CD147 is activated, and the p50 and p65 subunits of NF-κB translocate to the nuclear envelope [73, 128]. CD147 activates inflammation in some cells, including macrophages, which inflammatory diseases may be triggered by them. In addition to increasing the expression of MMP-9 and the production of proinflammatory cytokines and chemokines in endothelial cells [73, 129]. SARS-CoV-2 entry receptors, such as CD147 and ACE2, are believed to activate the NLRP3 inflammasome (nucleotide-binding domain, leucine-rich repeat, and pyrin domain-containing protein 3), which induces cleavage of IL-1 and IL-18 cytokines [73, 130].

### Targeting spike protein by using lectins and lectibodies

**Fig. 5:**
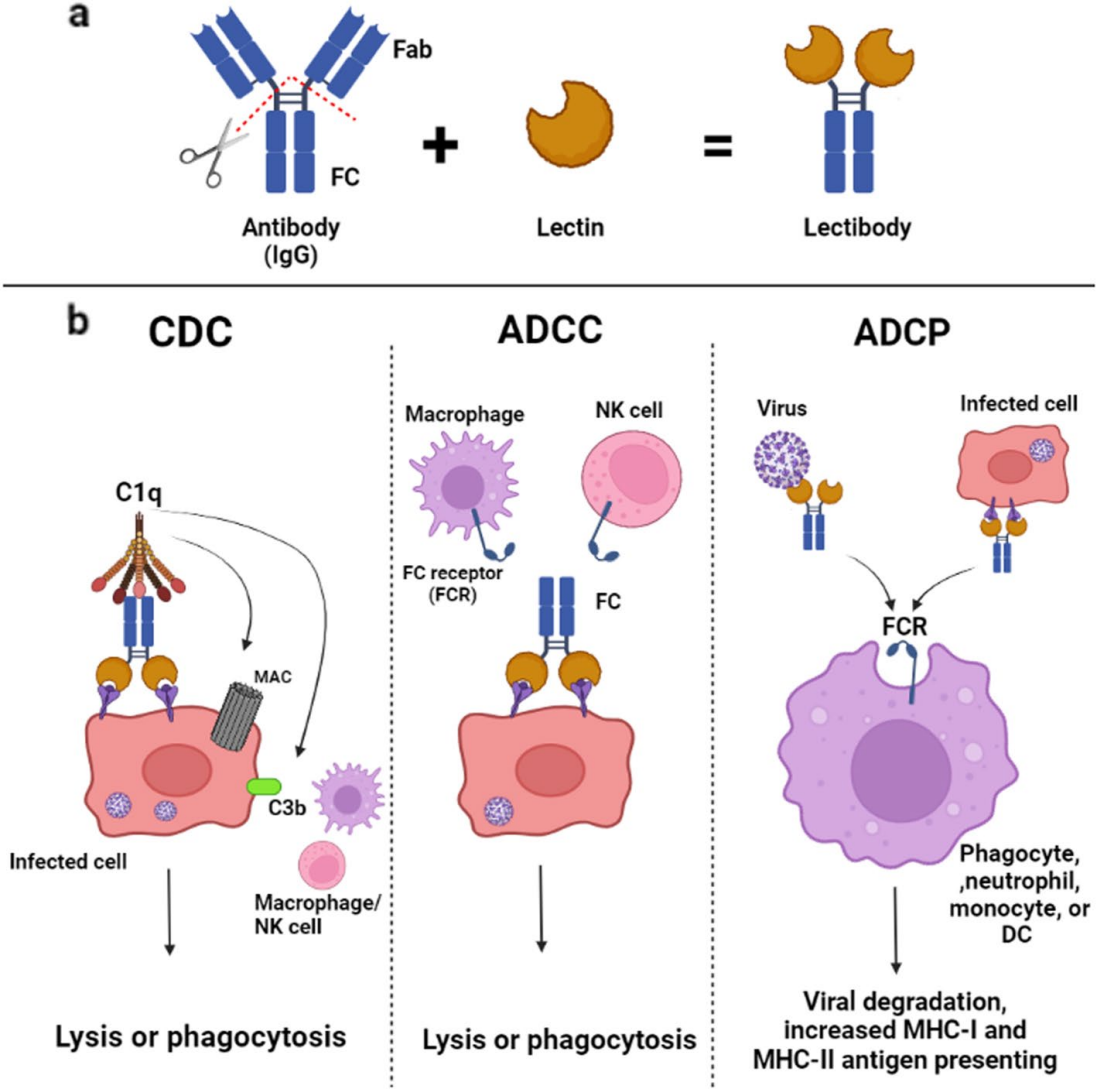
(**a**) A compound known as a “lectibody” is created when the IgG crystallizable Fc and lectin of an antibody are combined. (**b**) Through the actions of CDC, ADCC, and ADCP, lectibodies’ lectins bind to surface glycoproteins, neutralizing viruses or infected cells and assisting the innate and adaptive immune systems in combating pathogens. Reprinted with permission from ref. [138]. Copyright 2022, Springer Nature

SARS-CoV-2, like other viruses, is contained in a glycoprotein envelope [131]. It also contains a glycoprotein that raises the possibility of using lectins as therapy [132]. During budding, the virus forms a double-layered envelope so that each of its components is dependent on the cell membrane from which it originated [133]. Some proteins present in SARS-CoV-2 envelope layers are glycosylated by host enzymes. These glycoproteins help develop and control immune system responses as well as virus attachment, invasion, and entry [134]. The S1 and S2 subunits each have 22 possible N-glycosylation sites and three potential O-glycosylation sites. Due to C-type lectin receptors (CLRs) preferentially binding specific glycans in a C-type lectin-dependent manner, the S1 glycoprotein of SARS-CoV-2 has ligands for many innate immune receptors [135]. Macrophage mannose receptor, macrophage galactose lectin, lymph node-SIGN, dendritic cell-SIGN, and dectin-2 are examples of CLRs that are often expressed by cells of the immune system such as macrophages and DC [136]. Lectins are not considered effective antiviral agents due to their cytotoxicity, mitogenicity, pro-inflammatory properties, small size, poor stability in the body environment, susceptibility to proteolytic lysis, and challenges in mass synthesis [137]. Lectibodies, modified lectins, have been developed using a variety of protein engineering techniques to circumvent these challenges [138]. A lectin and the crystallographic fragment (Fc) of an immunoglobulin G (IgG) antibody combine to create a “lectibody” protein. As an antibody, this protein can bind carbohydrates (Fig. 5a) [139]. In addition to complement-dependent cytotoxicity (CDC), antibody-dependent cell-mediated cytotoxicity (ADCC), and antibody-dependent cell-mediated phagocytosis (ADCP), lectibodies are Fc-mediated antibody effectors that can bind to surface glycoproteins via lectins, neutralise viruses or virus-infected cells (Fig. 5b) [139]. While C3b binds to pathogens and infected cells to initiate phagocytosis and clearance of immune complexes, the release of C3 and C5 molecules recruits and activates effector cells of the immune system [140]. Infected cells lyse after the formation of a membrane attack complex. The Fc gamma receptors (FcγRs) on natural killer (NK) cells and the Fc domain of antibodies bound to viral antigens on infected cells interact to trigger the ADCC reaction (Fig. 5b) [139]. Cytotoxic granules are released and destroy the infected cells. Anti-Ebola monoclonal antibodies significantly affect NK cells through ADCC [141]. In previous studies, it was discovered that phagocytic cells ingest virus-antibody or antibody-infected cell complexes during ADCP. After processing, the antigen either binds to molecules on the cell surface of the major histocompatibility complex (MHC) or is transferred to lysosomes to be degraded. Mice treated with anti-SARS-CoV antibodies showed that ADCP reduced SARS-CoV infection [142, 143].

#### Membrane protein

The membrane (M) protein is 221 amino acids long and has little similarity with the M proteins of other coronaviruses [144]. This protein is important for the formation of virions inside the cell (between the ER and the Golgi body). The M protein consists of three structural components: 1) the N-terminal portion of the virion that protrudes from the membrane. The N-terminal domain, which is sensitive to protease, binds to the surface of the virus; 2) transmembrane domains; and 3) there are two domains in the C-terminal. The amphipathic domain near the transmembrane region of the third domain is followed by a tiny hydrophilic region that connects to the host viral or cytoplasmic membrane, where virus assembly and germination occur [145]. The cell membrane protein eventually becomes a site for the production of new viral particles in the host cell. In addition, the M protein is important for enhancing aggregation through interaction with viral ribonucleoprotein and S glycoprotein at the site of budding [146]. This protein in SARS-CoV has a protective glycosylated region that may be critical for host-virus interaction [147] and this is notable because it can inhibit NF-κB activity and reduce the production of COX-2 (an important inflammatory protein) [148]. It can interact with IkB kinase beta (IKKβ) and prevents the development of a fully functional IKK signalosome, thereby reducing NF-κB activity [149]. M protein can also interact with IKKβ and other subunits of the IKK signalosome. Another important point is that the M protein can bind to all structural proteins [150]. For example, the interaction of the M protein with the nucleocapsid (N) protein contributes to the stability of the N protein [151]. On the other hand, when the S protein and the M protein bind to each other, changes occur that may affect how the virus interacts with the host cell and enters the cell [152].

#### Nucleocapsid protein

One of the most common structural proteins of the SARS-CoV-2 virus is the N protein [153]. N protein, a multifunctional protein, is essential for transcription and replication [154]. This protein is required for the creation of ribonucleoproteins that regulate the replication and synthesis of the viral RNA genome [155]. The main function of the N protein is to bind to the RNA genome of the viral infection and package it into a long nucleocapsid, which is also known as ribonucleoprotein [156]. Most studies have shown that this protein affects host–pathogen interactions, including actin reactivation and host-cell cycle progression [157]. This protein is highly immunogenic and is present in large quantities during infection [158]. Inside the virus, the N protein protects and stabilizes the viral RNA [159]. During virus assembly, this protein assists viral membrane proteins and interacts with the M protein [44]. It also affects RNA folding, translation, and the cell cycle [157]. The N protein is associated with transcription and replication complexes in infected cells [160]. Evidence suggests that this molecule may play a role in the pathophysiology of CNS infections. It is hypothesized that the N protein may activate toll-like receptors (TLR)3, TLR7, or TLR8, and subsequent signaling pathways may increase the activation of NF-κB and NLRP3, leading to a cytokine storm and inflammatory responses [161]. As a result, they may play a role in the pathogenesis of a number of diseases such as the appearance of cancer, coagulation, neurodegenerative disorders, and cardiovascular diseases [162]. Researchers should be able to comprehend the contribution of each component of innate and adaptive immunity to COVID-19 infection with the assistance of the data on SARS-CoV and MERS that are already available [161]. Even though TLR7/8 is the only TLR that can recognize ssRNA, COVID-19-associated genetic material, other TLRs, like TLR3, TLR4, and TLR6, may also be involved in COVID-19 infection [161, 163]. The type of TLRs (agonist/antagonist) that are treated depends on the stage of the disease. TLRs and related signaling pathways should be studied, as they have been associated with viral susceptibility and lethality in other coronavirus families. A therapeutic target may be to reduce inflammasome activation and neutrophil trap development [161]. Many studies based on the TLR pathway are being conducted on COVID-19 [161]. Bioinformatics study may help us understand how TLRs interact with RNA and proteins of COVID-19 [161]. TLR3, TLR7/8, and TLR9 are all found on the endosome surface, while TLR2/6 and 4 are found in the cell membrane [164]. Pro-inflammatory cytokines and IFN-I are produced as a result of the activation of the downstream adaptive response of MYS88 and TRIF proteins [161]. MYD88 activates TRAF6 in the TLR2/6 and TLR4 pathways, but it also activates IRAK4 and, indirectly, TRAF6 and TRAF3 in the TLR7/9 and TLR9 pathways [161]. When TRAF3 is stimulated, IRF3 is then activated, and IFN-I is released [161]. Liquid–liquid phase separation (LLPS) occurred in RNA and N protein. The length and concentration of ssRNA determine the LLPS. With short ssRNAs, N protein makes typical droplets that look like spheres, but with long ssRNAs, it makes solid structures. Zn^2+^ could make LLPS better. The possibility that the N protein/RNA LLPS is necessary for the assembly of the SARS-CoV-2 virus provides insight into the development of intervention strategies to prevent the COVID-19 pandemic by disrupting the LLPS and viral assembly [165].

#### Envelope protein

The envelope (E) protein has 10–74 amino acids and occurs in monomeric and pentameric forms [166]. This protein is present in approximately 20 copies of viral material [167]. Previous research has shown that mutagenesis has a significant impact on the development and spread of viral infections [168]. In particular, viruses lacking this protein cannot infect the host cell and have a very low viral titer in the host cell [169]. This protein is located in the secretory pathways between the ER and the Golgi apparatus of the host cell [170]. The C-terminal region of the E protein is structurally located inside the envelope of the virus so that it is placed around the envelope and finally entrapped inside the envelope [171]. It can block the host cell's ability to replicate and spread the virus throughout the body [172]. Although its purpose is still unknown, this small protein can cause oil bubbles to form inside the virus [173]. It accompanied by the M and N proteins is very important for the development and propagation of virus particles in SARS-CoV-2 [174]. E protein interacts with host cell proteins and acts as an ion channel [169].

#### Hemagglutinin esterase protein

Infection requires hemagglutinin esterase (HE) protein, which can act as a second protein in the recognition of host cell surface receptors [175]. On the other hand, the HE protein of the human recombinant virus OC43 (HCoV-OC43) can affect the replication and viral infections of cells and is important for the dissemination of infectious agents [176, 177]. Two functional features of the HE protein are its affinity with sialic acid and its ability to enzymatically degrade the receptor on the host cell surface [177]. These two distinct functions of the HE protein may help the virus to enter or facilitate exit from the cell surface. In addition, It helps in the efficient creation of viral particles. This protein can also remove the S protein from 9-O-acetyl sialic acid on the cell surface, causing the virus to fuse with the cell membrane [178].

### Nonstructural proteins of SARS-CoV-2

#### Accessory proteins

There are two genetic subgroups in group 1 coronaviruses [179]. HCOV-NL63 and HCOV-229E are human coronaviruses belonging to groups 1a and 1b [180, 181]. The set of genes encoding one or more accessory proteins between the S and E genes includes HCOV-NL63, ORF4a, and ORF4b for HCOV-229E and ORF3 protein for PEDV [182]. ORF1ab occupies two-thirds of the entire genome and sub-genome to play a role in viral pathogenicity, eliminate its replication function and also plays a role in cell signaling and gene expression regulation [183]. For replication and production of the viral genome, this protein binds to 16 proteins. SARS-CoV-2 has six major ORFs: ORF1ab, ORF3, ORF6, ORF7a, ORF8, and ORF10 [184]. Two polyprotein precursors, pp1a and pp1ab, are produced by ribosomal translation of ORF1a and ORF1b using positive-strand RNA as a template [185]. To form the viral RNA replication-transcription complex, viral proteases first cleave these polyproteins into 16 NSPs, then partially translocate to the ER membrane [185]. CoV produces full-length sgRNAs and intermediate negative-sense RNAs that serve as blueprints for subsequent viral genomes [186]. The translation of the first four structural and supporting proteins is complete. N proteins produce nucleocapsids that contain the complete progeny genome [187]. Virions that produce envelopes enclose recently produced nucleocapsids. Virus particles are finally released from infected cells after being transferred to the plasma membrane by smooth-walled vesicles [188]. Double-membrane vesicles (DMVs), often found in the perinuclear region of the cell, are formed as a result of the CoV infection process, which engulfs the internal membranes of host cells [189]. DMVs create an environment that promotes viral RNA synthesis while protecting the ability of the innate immune system to recognize double-stranded RNA. Although the bilayer membrane origin of these autophagosome-like DMV is still unknown, specific studies suggest that the autophagy machinery is involved in their production. It is still unclear how autophagy contributes to the conversion of host membranes to DMV. Researchers are investigating the link between CoV infections and autophagy because of the structural similarities between the DMV and the autophagosome [190].

#### ORF3a

This protein is the largest accessory protein of the virus with 274 amino acids translated from ORF3a, which is located in the viral genome between the S and E genes [166]. These accessory proteins can be activated through the interferon (IFN) signaling pathway and release pro-inflammatory cytokines that can lead to changes in the infected cell's environment and cause inflammation, possibly leading to the most lethal symptoms of COVID-19 [191]. ORF3a also helps the virus to escape by creating a hole in the membrane of the infected cell. This protein is used to identify patients with COVID-19 in lung epithelial cells; the accessory protein, ORF3a, can increase the expression and secretion of fibrinogen. The pathogenesis of SARS-CoV-2 may be caused by increased fibrinogen and cytokine production. On the other hand, it may promote chemokine NF-κB, IL-8, and RANTES CCL5. It contains the primary binding site of caveolin-1. Caveolin is responsible for signal transduction. Since multiple signals bind to and control caveolin, it may play a role in cell cycle regulation. Finally, accessory ORF3a protein is a byproduct that helps produce new viruses as well as their escape from the host cell [192]. The virus selectively targets mitochondria, where it attacks and destroys them, depleting the cells' energy and reducing their capacity to fight infection, including through autophagy [193, 194]. By controlling ACE2 and ORFs, SARS-CoV-2 bypasses host cell defense mechanisms and accelerates virus replication. The mitochondrial ORF-96 protein of the virus damages mitochondria-related genes such as DRP1, MAVS, TRAF3, and TRAF6 [195]. ORFs like ORF3a can alter mitochondrial homeostasis (biogenesis, fusion, fission, and mitophagy) and function by focusing on the mitochondrial deubiquitinase USP30 [196, 197]. ORF3a protein also induces apoptosis in mitochondria. The BCL-2 families of neuroprotective proteins and Bax proteins, which can switch to initiate a cell death cascade, are in balance in the cellular homeostasis system. This can occur in response to extracellular stimulation by stress, viral infection, excessive immune cytokines secretions, etc. Even though the molecular structure of Bax is normally stable, when it constricts a virus, it translocates to the mitochondrial outer membrane, where it is deposited, releasing cytochrome c and initiating the process of apoptosis [184]. In addition, ORF3a protein increases the activity of truncated Bid (tBid), which causes mitochondrial perforation and promotes the release of apoptogenic compounds. The unique DNA of the degraded mitochondria is then released into the blood whose presence at high levels has now been reported to predict poor COVID-19 outcomes [197, 198]. Furthermore, in COVID-19 patients, ORF3a protein activates HIF-1, enhances viral infection, and increases cytokine production. Since HIF-1 controls inflammation and glycolysis, it is likely to be involved in the pathogenesis of COVID-19 [199, 200]. The transcription factor HIF-1 is induced by hypoxia. In cells infected with SARS-CoV-2, the increase of HIF-1 causes the production of A-disintegrin and metalloprotease 17 (ADAM17). This encourages TNF production, TNF processing, and innate immune cell uptake of processed TNF. ADAM17 triggers a cytokine storm by disrupting the IL-6/IL-6R/gp130 complex and converting IL-6R into a pro-inflammatory agent [201, 202]. The transcriptional activity of the HIF-1 protein may affect various processes, including the body's capacity to use glucose, metabolic pathways, cell growth, angiogenesis, and metastasis. HIF-1 can also alter the expression of genes related to cancer growth and autophagy [202]. Meanwhile, FLT3-ITD mutation in AML patients is associated with autophagy and HIF-1 activation [203]. The PI3K/AKT/mammalian target of rapamycin (mTOR) signaling is promoted by the FLT3-ITD mutation which is the most common mutation observed in AML [204]. The FLT3-ITD mutation, previously described in AML patients with this genetic aberration, can be seen as an upstream mechanism in HIF-1 activation, even though the mTOR pathway increases HIF-1 levels [205]. Furthermore, it has been noted that AML patients expressing FLT3-ITD have higher amounts of autophagy. Both treatment resistance and the development of AML with FLT3 mutations are associated with autophagy [206]. FLT3-ITD mutation enhances autophagy in AML cells via ATF4, prolongs the lifespan of leukemia cells, and induces tolerance to FLT3 inhibitors. In addition, the treatment of FLT3-mutated AML using autophagy inhibitors is more effective compared to FLT3-inhibiting drugs [207]. In addition, Deeb et al. found that in older AML patients with normal karyotypes, higher cytoplasmic HIF-1 expression was associated with worse prognosis after conventional therapy. Therefore, COVID-19-stimulated autophagy and HIF-1 could serve as markers of AML patients, especially patients with FLT3-ITD mutations. COVID-19 patients with AML who carry FLT3-ITD mutations are more likely to experience severe disease [208]. It is hypothesized that in addition to predisposing these patients to a severe course of COVID-19 and high mortality, COVID-19 and FLT3-ITD-dependent autophagy and HIF-1 overexpression may also play a role in progression of leukemia and drug resistance. Not to mention, we believe that FLT3 inhibitors and Not to mention, we believe that FLT3 inhibitors and drug interactions with autophagy could be a potential treatment for FLT3-ITD mutant patients with COVID-19 to reduce the risk of mortality, halt the progression of leukemia, and stop drug resistance. This also requires more research to confirm this claim [209].

#### ORF3b

ORF3b accumulates in the nucleus and mitochondria of infected cells [210]. This protein inhibits cell growth in the G0/G1 phase. On the other hand, apoptosis and necrosis are caused by the overexpression of this protein. Moreover, this protein affects how the host's innate immune system reacts to SARS-CoV infection. This protein inhibits the production and signaling of IFN, which is a key component of the antiviral immune response [211].

#### ORF6

It is a very small protein with a molecular weight of approximately 7 kDa, located in the Golgi and ER. The N- and C-terminals are composed of 2–37 and 54–63 amino acids, respectively. This protein is involved in reducing the synthesis of primary IFN and the functional signaling of IFN. It is the main antagonist of antiviral IFN and results in the suppression of IFN induction by multiple signaling molecules such as MDAS, MAVS, TBK1, and IFN regulatory factor 3 (IRF3)-SD [212]. It is important to note that the last amino acids of the DEEQPMEID tail are required for ORF6 function in the suppression of IRF3 and STAT1 activation [213]. On the other hand, this sequence could be a suitable choice for therapeutic applications, because a role restriction peptide can reduce the severity of SARS-CoV-2. ORF6 also interacts with NUP98 and RAE1, which form a nuclear pore network [214].

#### ORF7a

This protein enhances NF-κB and p38 while inhibiting cellular protein transport [215]. Research has shown that new viruses escaping from the host cell can be stopped by a protein called tetherin. In this case, a viral protein called ORF7a helps viruses escape by creating nicks in infected host cells that are the source of tetherin [216]. It can drive infected cells to commit suicide and help SARS-CoV-2 damage lung cells. It has been demonstrated that the coagulation proteins VKORC1, SERPING1, and PABPC4 interact with the SARS proteins [217]. Blood coagulation is influenced by ORF7a binding to VKORC1, which may be altered in individuals with specific VKORC1 polymorphisms [217]. VKORC1 is required to keep vitamin K levels active, which in turn keeps important clotting components active [217]. This may be related to the role of vitamin K in the synthesis of coagulation factors and proteins that control coagulation, the antagonistic interaction between vitamin K and inflammatory responses, or the antagonistic relationship between vitamin K and IL-6 levels [218–220]. In COVID-19 patients, the inflammatory and immune response plays an important role in the development of symptoms [221, 222]. Computational and experimental data support the binding of ORF7a and VKORC1. Some VKORC1 mutations affect pulmonary intravascular coagulation of COVID-19 [217]. A deficiency of coagulation factors and active vitamin K, which are essential for the carboxylation of coagulation factors, may result in severe damage and clotting in the lungs [223]. By preventing the conversion of vitamin K epoxide to active vitamin K, the interaction of ORF7a and VKORC1 may further reduce pulmonary hemorrhage [224]. This interaction may be less significant in warfarin-resistant individuals due to increased VKORC1 protein synthesis or altered VKORC1 structure, leading to increased clotting and worse prognosis in these individuals [217]. In addition, the SARS-CoV-2 ORF7a binds to immune cells (*i.e.*, HLA-DR, DP, and DQ) and induces significant inflammatory reactions in the peripheral blood of the patient (altering proinflammatory cytokines (*e.g.*, IL-1 and IL-6). Finally, it significantly reduces the level of HLA-DR, HLA-DP, and HLA-DQ molecules in monocytes. CD14 is one of the factors that indicate this stage of the process. Along with monocytes, other pro-inflammatory cytokines also show significant changes, suggesting that this protein may be important for the onset of the cytokine storm in COVID-19 [73, 223]. The viral component, which has an immunoglobulin-like structure and helps the virus to escape from the host's immune system, has a protective function for the virus [225, 226]. JNK is an important pathogenic mechanism for SARS-CoV [227]. By activating ORF3a, ORF3b, and ORF7a in this pathway, a large amount of pro-inflammatory factors are synthesized, which can cause lung damage and produce more pro-inflammatory factors [228]. The severity of the disease is associated with a hyperinflammatory syndrome characterised by fulminant and severe hypercytokinaemia with multi-organ failure and a cytokine profile similar to secondary haemophagocytic lymphohistiocytosis [229, 230]. Tumor necrosis factor, IL-2, IL-7, IFN-inducible protein-10, granulocyte colony-stimulating factor, macrophage inflammatory protein 1, and monocyte chemoattractant protein-1 are some of the proteins produced under these conditions [230, 231]. In addition, SARS-CoV-2 showed increased infectivity and transmissibility but lower mortality when compared to other respiratory syndromes coronaviruses, such as MERS-CoV and SARS-CoV. There may be a link between SARS-CoV-2's increased virulence and its stronger binding to ACE2 and mutations in its genome. ORF8 and ORF10 proteins, the NSP2 and NSP3 proteins, shorter 3b segments, deletion of 8a segments, and larger 8b segments are all mutated in the SARS-CoV-2 gene [230].

#### ORF7b

This protein differs from other proteins of the SARS-CoV-2 virus protein family because it lacks the same sequence [216]. The transmembrane domain of ORF7b is essential for protein retention in the Golgi apparatus. Alanine scanning assays have shown that the amino acids at positions 3–15 and 22–29 of this protein are critical for the maintenance of the Golgi complex. ORF7b of SARS-CoV-2 is 81% similar to this protein in SARS-CoV [232].

#### ORF 8a

ORF8a proteins, which contain a signal sequence, play a key role in ER insertion into the lumen of the ER. ORF8a interacts with a variety of host cell proteins involved in the ER-associated degradation pathway [233]. ORF8a is released from the ER lumen because anti-ORF8a antibodies are one of the most important markers of SARS-CoV-2 infection. In addition, SARS-CoV-2 patients release this protein, which affects the IFN-I signaling pathway. Conversely, cells with ORF8a produce less MHC-I [234]. The causes of immunodeficiency in COVID-19 may be determined by examining immune responses to SARS-CoV-2 over time. For instance, multiplex cytokine analysis and high-dimensional cytometry to fully assess the T and B cell fractions in the peripheral blood of COVID-19 patients recovered donors and healthy controls. This research includes immunological and clinical factors to detect temporal changes in the population of activated plasmablasts, effector memory T cells, and CD4^+^ follicular T cells. Three “immunity types” with different severity scores for COVID-19 were found using unsupervised cluster projection. Specifically, one sample showed significant activation of CD4^+^ T cells and plasmablasts associated with COVID-19, but the other sample showed minimal to no lymphocyte response. This research emphasizes different immune mechanisms at work in COVID-19, which could include changes in immunosuppressive responses [235]. Activation of MHC-I specific CD8^+^ T lymphocytes and eradication of infected cells depends on the presence of MHC-I antigens. Proteins degraded by the cellular proteasome complex are loaded onto MHC-I molecules by the ER and then transported to the cell surface, where antigen-specific CD8^+^ T lymphocytes recognize the proteins. Several viruses have developed the ability to prevent MHC-I processing and present viral antigens to infect and disseminate throughout the host [236]. Similar methods are used by SARS-CoV-2 to redirect MHC-I through viral proteins [237, 238]. MHC-I autophagy is induced by the SARS-CoV-2 ORF8a protein, which also resists CTL surveillance [237]. The SARS-CoV-2 ORF8a gene grew rapidly in the first three months of the outbreak. These isolates included some with a 382 nt deletion covering the ORF7b and ORF8a gene area, which is associated with a robust T-cell response and a favorable clinical outcome [239, 240]. These observations highlight the idea that variants of concern and ORF8a protein have evolved to improve their ability to downregulate MHC-I to prevent antigen-specific memory CD8^+^ T cells induced by past infection or immunization [241]. ORF8a interacts with IRF3 to trick the host's immune system and perhaps avoid recognition [242]. The interaction between ORF8a and IRF3 has been shown to target different elements of the IFN signaling cascade, inhibiting the host immune system and enabling the successful progression of infection [243]. Many studies have also identified defined conformational changes of ORF8a such as W45L, V62L, and L84S that can better evade the host immune system. ORF8a has also been shown to form intracellular aggregates in lung epithelial cells. It has also been found to induce stress in the ER, which supports the evasion of the immune response [244]. Therefore, targeting the ORF8a-IRF3 pathway is considered an important target for the development of new drugs against SARS-CoV-2 [245].

#### ORF8b

**Fig. 6:**
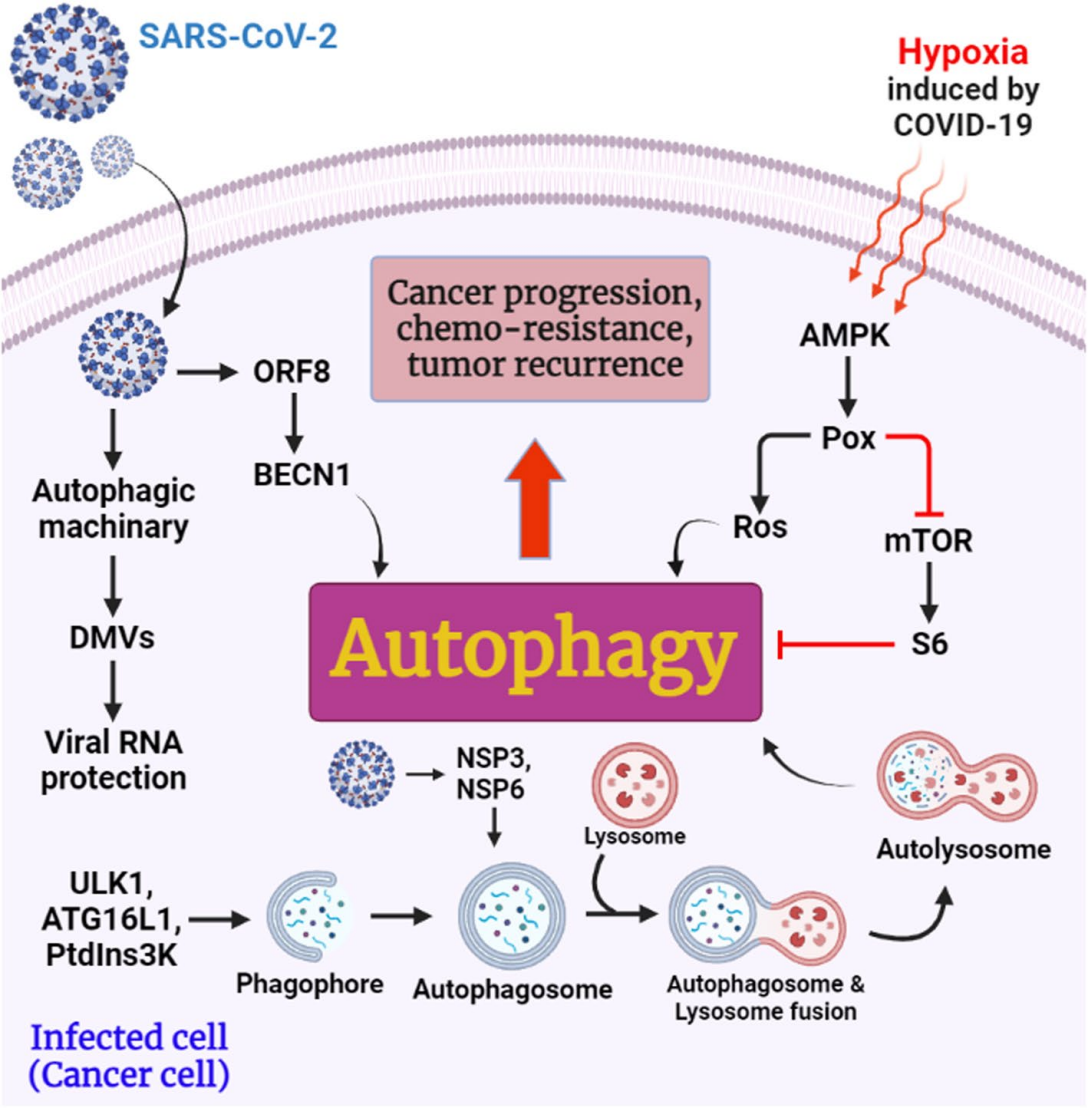
The potential contribution of SARS-CoV-2 ORF8, NSP3, and NSP6 to cancer growth, chemoresistance, and tumor recurrence in infected cells (cancer cells). Reprinted with permission from ref. [248]. Copyright 2022, Springer Nature

This affects the modification of the E protein and promotes virus replication, and may be important for limiting virulence [246]. ORF8b has an IgG-like structure and does not alter the ORF7 genome [247]. Interestingly, intracellular aggregates of SARS-CoV ORF8b induce cellular stress through activation of the EB transcription factor and its target genes, leading to increased autophagy (Fig. 6) [248]. However, excessive levels of ORF8b can damage lysosomes, interfere with autolysosomal homeostasis, and impair the cell's capacity to degrade cargo proteins [190]. Consequently, the propensity of viral ORF8b to the cluster may protect SARS-CoV from destruction. They also discovered that the ORF8 and SARS-CoV-2 N proteins of the mTORC1 pathway interact with La ribonucleoprotein 1, translational regulator (LARP1), and FK506-binding protein (FKBP) prolyl isomerase 7 (FKBP7), but not interact with each other [249]. Viral N and ORF8 proteins may induce autophagy, but further studies are needed to confirm this [190].

#### ORF10

This protein in SARS-CoV-2 has the most immune epitopes among all ORF proteins, which can be a suitable target for vaccine development [250]. This protein has a molecular recognition feature a region of 3 to 7 amino acids, which serves as a molecular recognition site for interaction with other proteins [251]. This is a critical feature of misfolded proteins that allows them to adapt to a variety of chemicals when they bind to different proteins and also allows them to interact with many proteins [251]. Previous research has shown that the virus uses bioinformatics to multiply components of the ubiquitin ligase complex, and the host machinery regulates ubiquitin for the virus. Notably, ORF10 of Pangolin-CoV-2020, a protein specific for SARS-CoV-2 and undetectable in SARS-CoV, shares 15–99% of the nucleotide sequence with ORF10 of SARS-CoV-2, which was labeled as a mysterious protein [251].

### Replicase proteins

To inhibit IFN signaling and the IFN-mediated antiviral response, the SARS-CoV-2 encodes some viral structural proteins and NSPs with different roles in viral replication and packaging [252]. The most important proteins are NSP1, NSP8, NSP9, and NSP16 because they limit protein transport, transcription, and translation in the host [253].

#### NSP1

This protein suppresses cell growth by interrupting the G-0 and G-1 phases of the cell cycle [254, 255]. It also binds to various ribosomal assemblies, including resting ribosomes, and translocates to pre-43S and similar pre-40S complexes, thereby inhibiting innate immune responses. Considering the importance of innate immune system cells in SARS-CoV-2 infection, NSP1 may be a suitable target for pharmaceutical agents and vaccines. The C-terminus of NSP1 serves as the main domain for interaction with ribosomes, which is required to control the cellular response to viral infection. Overall, NSP1 is a potential risk factor for coronaviruses and an attractive target for live attenuated vaccine development [256, 257].

#### NSP2

NSP2 proteins are the most variable NSPs in the coronavirus family [258]. Because NSP2 sequences are different in all coronaviruses. This protein works with the host to perform host functions and regulate infection and it is required for optimal virus replication. When NSP2 was deleted from the virus, the virus titer and the level of RNA synthesis were reduced on average compared to the wild type. This protein interacts with components of the cytoskeleton and plasma membrane of the primary site of virus production, as well as with vesicle components. A212, ZFANDs, NUMBL, USP15, STAT5B, IBTK, CYLD, and TRIM26 are a few other innate immune system elements that are associated with NSP2. It also associates with key proteins of autophagy such as WIPI1, WIPI2, and MAP1LC3B, as well as with important apoptosis regulators such as AVEN, BAG3, AREL1, TP53BP2, CASP8, and ZAK, and many kinases [259, 260]. Thereupon, this protein is associated with severe disruption of signal transduction in infected cells. On the other hand, NSP2 may play a role in altering the cellular response to infection and signaling death. NSP2 replicas are required for replication and can bind to inhibitors of apoptosis 1 and 2, leading to host cell survival. To maintain the functional integrity of mitochondria and defend cells against various stresses, this protein interacts with host PHB and PHB2 to alter the signaling system for host cell survival. The COVID-19 NSP2 protein has an entry pocket. A tight bond is now formed between nigellidine and CYS240, as well as an H-bond with LEU169, VAL126, TRP243, ALA127, CYS132, THE256, GLY257, TYR242, and VAL157 [261, 262]. Finally, it promotes mitochondrial integrity and alleviates cellular stress to keep the virus alive, and they play a role in viral replication [262, 263].

#### NSP3

**Fig. 7:**
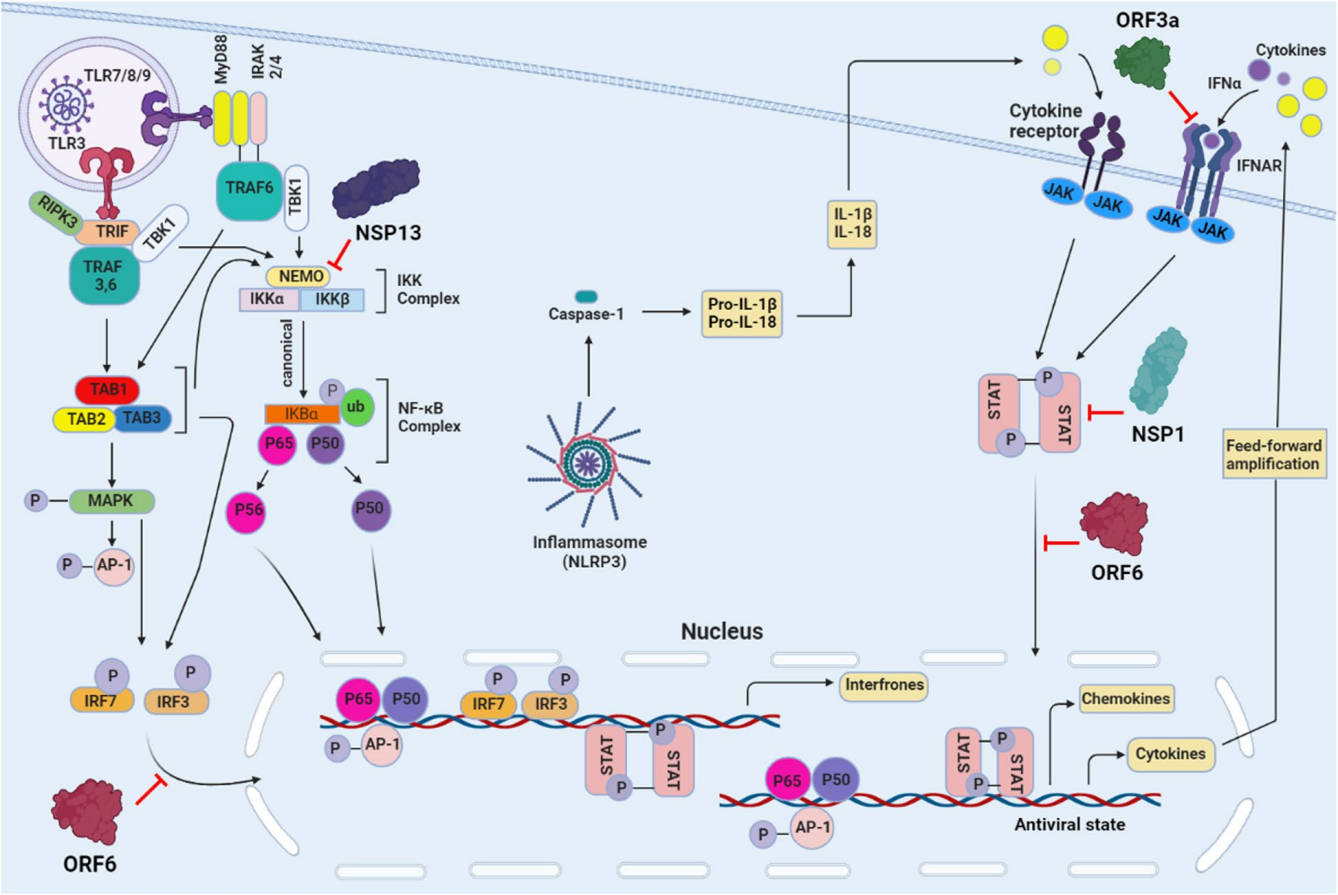
Intracellular signaling pathways stimulated by SARS-CoV-2 and the interactions of NSPs and ORFs with them

The novel coronavirus encodes a large and versatile protein with multiple domains [264]. Mac1 or X domain is one of the domains that can bind to ADP-ribose (ADPr) and act as a transcription factor [265]. Expression of NSP3 in macrophages activated by IFNλ indirectly regulates long-term expression dependent on STAT1 pro-inflammation through IFN-stimulated genes by inhibiting the reduction of STAT1 through MARylation and increasing the pool of STAT1 molecules available for phosphorylation [73]. This method helps to characterize cytokine storms in severe cases of COVID-19. In an interesting hypothesis, the IFN-stimulated gene ACE (SARS-CoV-2 receptor), which is associated with clinical symptoms and viral eradication, is thought to be present in many cells of the lung and small intestine [266]. For example, the discovery of STAT1 binding sites in the ACE2 promoter region by NSP3 in IFN-stimulated macrophages may lead to overexpression of SARS-CoV-2 receptors on the surface of auditory epithelial cells and prolong the duration of infection. By infecting more individuals and releasing more IFN, it increases STAT1 activity. This mechanism leads to increased inflammation and viral receptors. It also neutralizes PARP14-dependent MARylation of STAT1 continuous production. Consequently, it prevents the control and termination of the pro-inflammatory phase. MARylated STAT1 can also prevent viral pathogenesis by balancing NSP3 protein activity. STAT1 is the expected NSP3 target of SARS-CoV-2 in all cases. Physical contact exists between STAT1 and the N-terminal domain of the NSP3 protein of SARS-CoV-2, a multifunctional protein that functions as a viral protease and can inhibit IFN responses [267]. STAT1-dependent ISG synthesis is inhibited by SARS-CoV-2 NSPs (*i.e.*, NSP1, NSP3, NSP6, and NSP13) by reducing STAT1 phosphorylation and reducing inflammatory conditions. ORF3a and ORF7b inhibit STAT1 function. NSP6 and NSP13 together with ORF7a and ORF7b inhibit STAT2 phosphorylation [268]. By binding to karyopherin-2, ORF6 prevents STAT1 from translocating to the nucleus [269] (Fig. 7). STAT1 nuclear translocation is inhibited by SARS-CoV-2 3CLpro, and STAT1 protein levels and STAT1-induced IFN phosphorylation are enzymatically reduced. It has also been suggested that 3CLpro may promote STAT1 autophagy. Increased expression of 3CLpro promotes viral replication by downregulating IFN and ISG signaling pathways [15]. PLpro can use the JAK/STAT pathway to de-ubiquitination and de- ISGylation. Its ability to precisely cleave STAT2 and block IFN signaling has been demonstrated [268, 270]. The N protein, one of the structural elements of SARS-CoV-2, has been shown to inhibit STAT1 and STAT2 phosphorylation and nuclear translocation, thus preventing the inflammatory cascade induced by the IFN-I pathway. In addition to hyperinflammation, the severity of the disease is also affected by coagulation [271]. In order to prevent thrombotic effects in in people with metabolic and cardiovascular diseases, the control of oxidized phospholipids caused by oxidative stress (OxPLs) in monocyte and endothelial cells has been studied [272]. There are many pathways to block the ACE2 receptor, inhibitors of TMPRSS2 (*e.g.*, camostat mesylate), drugs that target STAT1 (*e.g.*, emapalumab), which are anti-IFN-γ baricitinib and ruxolitinib, two JAK1 and JAK2 inhibitors that block ACE2-mediated endocytosis, antibody monoclonal that targets the S protein. Furthermore, PARPs that block antiviral ADP-ribosylation can help with treatment [268].

#### NSP4

This protein interacts with other proteins and causes fluid bubbles to form in infected cells. In addition, parts of new forms of the virus are created inside these bubbles [273]. This protein together with NSP3 and NSP6 forms a complex that is important in viral replication by inducing DMVs. This protein destroys MAVs and controls NOTCH1 degradation. It ubiquitinates the chemokine receptor CXCR433 and directs it to the destructive pathway of HGS and STAM, both components of the ESCRT complex. NSP4 has also been shown to interact with the dual effects of the ACE2 receptor and control its neutralization. Altogether, through interaction with these machines, it appears as a major regenerator of the host membrane, and in SARS-CoV-2 infection, it disrupts two homeostases of methylated proteins in the membrane [260]. Multiple interactions occur between SARS-CoV-2 proteins and host proteins. The most interactive viral proteins were E, M, NSP4, and ORF7b, which frequently interacted with host proteins involved in immunity and translation in all organ types. APOB, PCDH9, and NCAM1 were the top three genes with the most virus-host protein interactions, all of which are consistent with symptoms of COVID-19 such as decreased apolipoproteins, decreased endothelial barrier function, and decreased immune cell proliferation. Finally, viral proteins have been investigated to interact with host genes involved in cardiomyopathy and angiogenesis [274].

#### NSP5

This protein has three domains: D1, D2, and D3, which are unique. Domains 1 and 2 contribute to the activation of the 3-chymotrypsin-like protease (3CL protein). The 3CL protein from NSP5 known as M pro is one of the best drug targets. Autodegradation of NSP5 leads to the formation of a mature 3-chymotrypsin product, and further degradation of this protein culminates in the formation of eleven non-structural products known as NSP4/16. Substrate transfer is between the first and second domains. For the protein to be catalytically controlled, protein dimerization is essential. Glucose molecules make up the remaining portion of the M protein structure, which is crucial for the dimerization and formation of a binding substrate. On the other hand, two amino acids Cys141 and His41 act as catalysts in the active site of the protein. Recent studies have shown that methods can be used to produce drugs resistant to M pro-SARS-CoV-2 [275]. The major protease of SARS-CoV-2 NSP5 inhibits both RIG-I and mitochondrial antiviral signaling (MAVS) protein. NSP5 selectively truncates the N-terminal 10 amino acids of RIG-I, preventing MAVS activation while increasing MAVS ubiquitination and proteosome-mediated degradation. NSP5 thereby prevents the induction of IFN in an enzyme-dependent manner by double-stranded RNA (dsRNA). A synthetic small molecule inhibitor blocks SARS-CoV-2 NSP processing and NSP5-mediated cellular degradation of RIG-I and MAVS, reinitiating the innate immune response and halting SARS-CoV-2 replication. This study provides a new understanding of how SARS-CoV-2 tricks the immune system of COVID-19 and suggests a potential antiviral drug [190].

#### NSP6

NSP6 is a membrane protein approximately 34 kDa in size with six membrane helices whose C-terminals are highly protected and enter the host ER membrane. A ring of small membrane vesicles surrounds the microtubule-organizing core (MTOC) after the transfection of NSP6. Since NSP6 leads to the formation of smaller autophagosomes, this protein may slow the proliferation of these structures. This protein may transport ER-regulated proteins produced by the ER and self-lyse autophagosomes in response to an immune response. When cells or organs fail, viral proteins typically lead to DMV double membranes (*e.g.*, ER stress, Golgi fragmentation, and changes in the autophagic apparatus). Consequently, the virus uses these proteins to hide and translate its so-called accessory proteins [276, 277]. The mouse hepatitis virus (MHV) model virus is often used in laboratories around the world, unlike the three novel hCoV viruses, which have limitations for careful study. MHV infection leads to various forms of autophagy activation, which are essential for the viral life cycle. In mouse embryonic stem cells lacking the autophagy-related gene ATG5, Cottam and colleagues found that MHV NSP6 could stimulate autophagic flux to form ER autophagosomes via omegasome mediators, but MHV could not form DMVs in these cells [278]. Compared to cells expressing ATG5, MHV replication is reduced more than 1000-fold in ATG5/embryonic stem cells, demonstrating that autophagy is critical for both DMV development and MHV replication [279]. A link between human coronavirus development and host cell autophagy has been proposed as a model. Emerging human COVIDs (SARS-CoV, MERS-CoV, or SARS-CoV-2) alter autophagy in host cells at different stages of infection: (A) SARS-CoV and SARS-CoV-2 NSP6 proteins induce omegasome intermediates in the initiation of autophagy (Fig. 6). To promote the formation of phagophores, all three CoVs can activate the ULK1 complex through the AMPK/mTOR pathway. (B) To prevent vesicle formation, MERS-CoV and SARS-CoV-2 degrade BECN1 via AKT1/SKP2. However, BECN1 is deubiquitinated by SARS-CoV and MERS-CoV PLpro to promote the initiation of autophagy. (C) SARS-CoV S proteins and NSPs induce ER stress that activates the unfolded protein response (UPR) and promotes phagophore elongation through the ATG5-12-16L complex. (D) Autophagosome-lysosome fusion limits autophagosome maturation by promoting insufficient autophagy inhibited by SARS-CoV and MERS-CoV PLpro. Meanwhile, SARS-CoV ORF8b can also damage lysosomes [190].

#### NSP7-8

NSP7-8 acts as a cofactor for SARS-CoV-2 RDRP (RNA-dependent RNA polymerase), also known as NSP12, which is an essential part of the replication and transcription machinery [280]. NSP12 alone has the lowest activity rate, while the maximum activity rate is determined by the common elements NSP7-8. These proteins are not sufficient for NSP12 to function properly. However, the combination of the two increases SARS-CoV-2 RNA synthesis by NSP12 [281].

#### NSP8 primase

This protein acts on the one hand as a primase for the synthesis of a primer for NSP12 and on the other hand in the synthesis of the RNA genome that is associated with the SARS-CoV-2 RNA virus. The protein also forms a small channel in the nucleus of an infected cell and can even cause molecules to move through the nuclear membrane, but no reliable information is yet available [282].

#### NSP9

NSP9 are binder dimers and are composed of a unique type that binds to each other through the GXXXG non-complex non-crossover pattern. The primary amino acid sequence of this protein in SARS-COV-2 is 97% related to the SARS-CoV virus, which is essential for virus replication. This protein is stable for single-stranded DNA and RNA oligonucleotides [283]. Through the use of reverse genetics, it was discovered that the SARS-CoV NSP9 gene could be altered to block the virus from spreading [284].

#### NSP10

Antiviral proteins (IFN, TNF, IL-12) present in human cells can destroy viral RNA. NSP10 contains viral genes that protect host antiviral proteins from damage. Nuclear elements involved in chromatin remodeling and mRNA processing directly interact with this protein. In addition, NSP10 stimulates NSP14 and NSP16 3′-5′ exoribonuclease (ExoN) and 2'-O-methyltransferase (2'-O-MTase). According to one theory, NSP10/14 may be a simple modification and amplification system [285]. During viral replication, NSP10/14 may be active. Its involvement begins when it corrects the mutated ribonucleotides produced by the RNA polymerase and allows it to resume its activity [286]. NSP10/16 is also essential for virus survival and replication. This complex codes for 2'-O-MTase, which helps the virus hide from the host's innate immune system by altering its genetic material to mimic the host cell's (human) RNA [287]. This allows the virus to multiply rapidly in the human body. The development of a therapy that destroys the SARS-CoV-2 NSP10/16 complex helps the immune system recognize and eliminate the virus. The SARS-CoV-2 genome was modified by the NSP10/16 complex. When the protein that acts as its activation complex, NSP10, is present, the SARS-CoV NSP16 protein is functionally activated and produces the NSP10/16 complex [288]. The viral genome is genetically modified to mimic human mRNA and protect it from the host's immune defenses [289]. This molecule binds to NSP14 to form further complexes, but also requires a cofactor. Before NSP10/16 RNA 2'-O methylation, which is responsible for RNA cap methylation, guanine N7-methylation occurs mediated by NSP14 [288, 290].

#### NSP12 polymerase

This protein contains seven motifs [291]. Among these, motifs A-F are highly protected from RDRP encoded by the virus [292, 293]. Motifs J, as an RDRP, are primer-dependent in several viruses. Motif C contains important catalytic residues (759 to 761 (SDD)) that are joined together by two adjacent strings in the β-turn [294, 295]. Motif F forms a fingertip that protrudes from the catalyst chamber and interacts with the finger extension and thumb subdomain rings [295, 296]. On the other hand, segmented negative-strand RNA virus (sNSV) polymerases (*e.g.*, influenza and bunya virus) need to be attached to a protected hook 5′-RNA protected to activate synthesis with fingertip, which is otherwise very flexible in the apo form [297, 298]. In the structure of coronavirus polymerase, the fingertip ring is fixed adjacent to the finger-extension rings by interactions with the NSP7/8 heterodimer. If this is not a heterodimer, the ring finger extension shows considerable flexibility which in turn destabilizes the finger-tip motif resulting in poor performance NSP12 which is why heterodimers NSP7/8 on top of subdomain thumb RDRP linked and sandwiches the finger-extension rings between them to stabilize its structure. Also, this protein is expressed in *Escherichia coli* and can modify the genetic letters within the new viral genome. Remdesvir is an antiviral drug that interacts with NSP12 in other coronaviruses [297, 298].

#### NSP13 helicase

When SARS-CoV-2 infects host cells, a variety of multivalent RNAs can be generated, and the virus can also assemble viral NSPs required for viral genome replication and transcription. Of sixteen NSP proteins related to SARS-CoV-2, four types of NSP have been discovered [299]. Among SARS-CoV-2 families, the NSP13 helicase is a key protein for viral replication and has the highest sequence conservation [300]. The main function of this protein is to prevent the virus from dying. In contrast, NSP13 converts double-stranded DNA into two single-stranded RNAs suitable for replication [301]. Deoxyribonuclease and ribonucleotide triphosphatase can also be hydrolyzed by this enzyme. This protein is also associated with centrosome proteins. SARS-CoV-2 NSP is an antagonist of IFN signaling [214]. The sequence of this protein is 99.8% identical to the SARS-CoV helicase sequence [302]. The terminal part of NSP13 is predicted to form a Zn^2+^ cluster that is immune to coronaviruses and nidoviruses [303]. NSP13 has NTPase activity and uses the energy of ATP hydrolysis to fuse base pairs. It is thought to be critical for RNA-related activities such as transcription and translation [304]. However, this critical enzyme is a suitable target for drug development against SARS-CoV-2 [304].

#### NSP14

This protein is changed by the coronavirus and is released by the COV3L protein. In other words, this protein can contribute to some genetic changes in viral RNA associated with NSP15 protein through its enzymatic activity. Because NSP12 copies the coronavirus genome, it sometimes adds new nucleotides to form new versions. The NSP14 function corrects these errors and can replace the incorrect nucleotide with the correct one. On the other hand, SARS-CoV-2 has developed the capacity to limit host RNAs through some pathways to enable successful replication in the host cell. The N-terminal region of NSP14 also contains the N7 methyltransferase (N7-MTase) enzyme, which is present near the end of the C-terminal of NSP14 and has an ExoN domain [305]. The mechanism of RNA capping plays an important role in the virus escaping from host immune cells and failure in capping RNA causes RNA viral destruction and ultimately, prevents the virus from replicating. Therefore, inhibition of the SARS-CoV virus may be possible by targeting the N7 region of MTase [306, 307]. The 3′ to 5′ ExoN and guanine N7-MTase activities of the NSP14 proteins of the coronavirus are well known. RNA polymerase dependent on viral RNA is believed to add mismatched nucleotides and the N-terminal domain of ExoN acts as a proofreader to facilitate the removal of these nucleotides [308, 309]. The proofreading activity of the ExoN domain is essential to maintain high levels of replication fidelity because coronaviruses have enormous viral genomes [310, 311]. Recently, changes in ZF motifs and active regions of the ExoN domain caused SARS-CoV-2 and MERS-CoV to exhibit a lethal phenotype [306]. An S-adenosylmethionine (SAM)-dependent N7 MTase is present in the C-terminal domain of NSP14 and is required for 5' capping of viral RNA. The 5' cap prevents recognition of viral mRNA by the host's natural antiviral defenses and promotes viral mRNA stability and translation [312]. NSP10, a zinc-binding protein with no reported enzymatic activity, and SARS-CoV NSP14 form a protein complex. NSP10 enhances ExoN activity but not N7-MTase activity when it interacts with the N-terminal ExoN domain of NSP14. Notably, SARS-CoV develops a lethal phenotype in response to NSP10 mutations that disrupt NSP10/14 junction. Like SARS-CoV-2 infection, SARS-CoV infection limits the synthesis of host proteins [313]. NSP1 overexpression reduces protein synthesis in cells, which supports previous studies on SARS-CoV and more recent studies on SARS-CoV-2 [313]. Furthermore, it was found that overexpression of NSP14 results in an almost complete cessation of cellular protein production. Mutations that inactivate either ExoN or N7-MTase enzymatic activities reverse translation inhibition mediated by NSP14 [313]. ExoN or N7-MTase mutants with a lower enzymatic activity completely abolish NSP14-induced translational inhibition. Moreover, the NSP10/14 protein fusion enhances the capacity of NSP14 to inhibit translation [313]. However, this increase in activity can be reduced by changing specific interacting residues. NSP14 inhibits the induction of IFN-dependent ISGs, which suppress the production of antiviral proteins. The ability of this translational inhibitor to reduce IFN-I responses [314]. This research led to the identification of the translational inhibitor SARS-CoV-2 NSP14, which is encoded by the virus and blocks the production of host proteins, including antiviral proteins [315]. It is essential to comprehend the processes by which SARS-CoV-2 undermines host immune responses in order to build next-generation antivirals and get ready for forthcoming viral infections [313].

#### NSP15

One of the mysterious enzymes related to NSP15 is a nidoviral RNA uridylate-specific endoribonuclease (NendoU) which carries the catalytic domain C-terminal and belongs to the family EndoU. Enzymes EndoU is responsible for various biological functions related to RNA processing RNA [316]. Furthermore, This protein can act as the hypothesis and probabilities. At first, this protein was directly involved in viral replication. Later it was shown that coronavirus viruses survive in the absence of this protein and perform their replication independently, leading to doubts about the role of the enzyme EndoU in the process of RNA. Recently, the EndoU activity of NSP15 is responsible for the interaction of the protein with the innate immune response. There is also the hypothesis that this protein destroys the genome of the virus to hide it from the host defense. However, NSP15 in the biology of the SARS-CoV-2 virus is essential [316].

#### NSP16

This protein forms a heterodimer with the NSP10 cofactor and enhances the activity of 2'-O-MTase. In addition, the genetic material changes the virus to look like human RNA. This blocks MAD from recognising the viral RNA and stops the innate immune response, both of which are essential for reducing coronavirus replication and infection. Therefore, the immune system will be better equipped to identify and get rid of the virus more quickly if a medication or chemical molecule is developed that restricts the activity of NSP16 [317].

### Vaccines for the SARS-CoV-2 virus

Using a wide range of technologies, including live attenuated, viral vector, DNA/RNA-based vaccines, protein-based vaccines, and inactivated vaccines, more than 100 vaccine candidates are currently being developed by industry and academic institutions [58, 318]. A complete vaccination should provide immediate, comprehensive, and long-term protection by preventing serious illness, hospitalization, and death [319]. Businesses and academic institutions are currently working on the development of more than 100 vaccine candidates using a range of technologies, including live attenuated vaccines, viral vector vaccines, DNA/RNA-based vaccines, protein-based vaccines, and inactivated vaccines. DNA- and RNA-based platforms have the best chance of achieving the fastest production speeds because their synthesis does not require fermentation or cultivation. DNA-based vaccines have other advantages. Since the vectors used only encode and express the target antigen and do not replicate, they are notable for their safety profile. The problem with viral vectors is that they cannot change back into a form that causes disease. Another interesting option is RNA-based vaccines, which have a low cost of production and strong safety records in animal tests [320]. Due to the possibility of low-cost manufacturing and the lack of vector-specific immunity, DNA-based vaccines are also a promising alternative [321]. These products can be used in prime and booster regimens with different products intended for the same patient because they lack vector-specific immunity (Inovio business). In addition, RNA-based vaccines such as mRNA-1273 (Moderna) and BNT162 (a1, b1, b2, and c2) from BioNTech SE/Pfizer, among others, are on the market [321] (Table 2).

#### Viral vectors

Vectors based on viruses are an effective tool for vaccination. Their capacity to infect cells gives them the ability to be highly efficient, specific, and capable of inducing strong immune responses [320, 321] (Table 2).

##### Inactivated vaccines

Inactivated vaccines are made using bacteria or viruses that have been inactivated by heat, chemicals, or radiation. These methods prevent the virus from multiplying, which makes them more stable and increases their level of immunity. These attributes allow for their use in immunocompromised individuals [320, 321] (Table 2).

##### mRNA-based vaccine

Recently, an mRNA-based vaccine (mRNA-1273) (NCT04470427) has been developed to prevent COVID-19, which evaluated the safety and immunogenicity of this vaccine for up to 2 years after the administration of the second dose. A comparable vaccine being developed by Curevac is still in pre-clinical testing [56, 322] (Table 2).

##### Subunit (recombinant protein) vaccines

Recombinant protein subunit vaccinations, an infectious virus do not require treatment; adjuvants can be used to boost immunogenicity [322, 323] (Table 2). For example, SCB-2019, a protein subunit vaccine candidate containing a stabilised trimeric form of the spike (S) protein (S-Trimer) combined with two different adjuvants that increased to achieve neutralizing antibody titers after two vaccinations. Convalescent serum samples from COVID-19 patients had similar responses. Antibodies to S-Trimer S protein component and its receptor-binding domain are directly correlated with this neutralizing activity [324, 325].

#### Live attenuated vaccines

live attenuated vaccines are produced by either employing a non-virulent strain or by creating a weaker strain of the virus. Numerous methods, including repeated passages in cultured cells and genetic alterations, can attenuate a virus. The ability to give live attenuated vaccines intranasally, which closely resembles a genuine infection and triggers mucosal immune responses, is a significant advantage [323] (Table 2).

##### Replication-incompetent vectors

A large number of vaccines in development are replication-incompetent vectors. These vaccines are typically based on other viruses whose genomes have been altered to express the spike protein and destroyed to prevent them from replicating in vivo [326]. Although modified vaccinia Ankara (MVA), human parainfluenza virus vectors, influenza virus, adeno-associated virus, and Sendai virus are also used, adenovirus (AdV) vectors account for the majority of these strategies.ChAdOx1 nCoV-19, developed by Janssen using an AdV26-based vector and developed by CanSino using AdV5; A candidate from the Gamaleya Research Institute (Ad5/Ad26) is also undergoing phase III clinical trials, while another from ReiTheram (gorilla AdV) is undergoing phase I clinical trials [327] (Table 2).

##### Replication-competent vectors

Attenuated or vaccine strains of viruses that have been modified to express a transgene, in this case, the spike protein, are typically the sources of replication-competent vectors. Animal viruses that do not spread quickly and do not infect humans are sometimes used as well. Because the vector is spreading in the person who has been vaccinated and frequently also elicits a robust innate immune response, this method can lead to a more robust immune response [328] (Table 2).

Information on structural and non-structural viral proteins can be used to design valuable diagnosis tests and vaccines. This matter could have many advantages for the global health system in the new coronavirus pandemic that could lighten disease complications by suggesting a new rapid test for diagnosis or effective vaccine candidates [329]. Therefore, the emphasis on research on genome structures, genes, and produced proteins can lead to identifying a proper target for laboratory diagnosis or vaccines for controlling viral infection. For the last purpose candidates for the SARS-CoV-2 vaccine are divided into two groups: 1) Vaccines that are based on genes, including DNA and messenger RNA vaccines, and vectors of recombinant vaccines and live viral vaccines because Antigens are produced in host cells. 2) protein-based vaccines that include completely inactivated viruses, or vaccines that use protein subunits [330]. For example, Lianpan et al. have shown that the S protein binding site with the specific name RBD is an attractive target for vaccine preparation despite limited immunogenicity. However, variable forms of RBD could overcome this limitation. This RBD vaccine can significantly increase neutralizing antibody (NAb) titers compared to its normal form (monomer) and protected against MERS-COV, COVID-19, and SARS infections [331]. In an interesting study by Salman Khan et al. structural proteins, S and E proteins, and non-structural proteins, ORF3 and ORF5 linked together by beta-defensin which have potency and are highly antigenic have been studied. The vaccine was designed for further expression transferred to *E. coli*. This vaccine will not only be effective in immunizing the population with MERS-CoV but also will be effective in immunization against COVID-19 [332]. Furthermore, four epitopes were selected from the S1 structural protein domain with 14 to 685 amino acids. The analysis showed that three epitopes of the selected epitopes are in the N-terminal domain of the S1 protein and one epitope is related to the binding part of the receptor. Consequently, this particular segment could be a good target for designing an antiviral vaccine [332]. Another study by Naz, et al. reported that structural S protein plays an important role in inducing neutralizing antibodies and T cell responses as well as protective immunity during infection. Therefore, the researchers, considering the importance of this protein in immunogenicity, named it a suitable candidate for a vaccine against host receptors (ACE2). On the other hand, vaccines made based on S protein, could be potential therapeutic targets against the SARS-CoV-2 virus, as it is likely to block the virus from interacting with the host receptor of the two host cells. Prevent pulmonary vascular permeability [333]. For example, Ong et al. developed reverse vaccination tools Vaxign revers, and the newly developed Vaxign-ML medicine. By examining the SARS-CoV-2 proteins, two or six proteins, including the S protein and five non-structural proteins (NSP3, 3CL-pro, and NSP10) predicted as an adhesive, which is essential for virus binding and attack on the host. Structural and non-structural proteins (NSP3, S protein, and NSP8) predicted by Vaxign-ML with high protective antigenic properties. The NSP3 protein contains the epitopes of MHC cells one and two T cells also predicted. Linear B cell epitopes are translated in specific locations and functional domains of the protein. These proteins can be suitable targets for the preparation of an effective COVID-19 vaccine [334]. For example, Yang et al. showed the recombinant vaccine made from the 319–545 locus of the RBD S protein receptor induced a potential functional antibody. The serum of immunized animals with this vaccine contains a high titer of antibody that blocks RBD binding to the ACE2 receptor. Inducing the antibody response by this vaccine activates several immune pathways and T cell lymphocytes (CD4). These findings demonstrate the importance of RBD in vaccine preparation for the prevention and treatment of SARS-CoV-2 [335]. For example, Lei et al. created a recombinant protein by binding the second extracellular receptor ACE2 of humans to the FC region of the human immunoglobulin IgG1. This protein has a high affinity for the RBD of SARS-CoV and SARS-CoV-2 and could have potential applications in the diagnosis, prevention, and treatment of this virus [336].

## Conclusion and future directions

The COVID-19 epidemic has been a serious threat to human society and the global economy since the beginning of the 21st century. However, there is currently no definitive treatment and only a few FDA-licensed COVID-19 vaccines. The introduction of safety informatics approaches in vaccine production has led to a huge revolution. Utilizing proper protein antigens can stimulate both the antibody response and the immune response triggered by the host. The immune response to common illnesses is limited but can be enhanced by the development of an epitope-based vaccine. Therefore, judicious selection is required to isolate the elements that are essential for the desired immunological response. Efforts are underway to find acceptable T-cell epitopes and to develop effective techniques for delivering such epitopes. The advantages of developing epitope-based vaccination include increased safety, reduced time, and the ability to tailor epitope combinations for better resistance. It also makes it easier to focus on the required immune responses to protected antigenic epitopes. The key epitopes of this virus include structural and NSPs, which can be excellent and effective candidates for making vaccines and treatments. It is also known that COVID-19 can lead to the development of several complications and diseases such as neurological diseases, cancers, etc. Hence, therapeutic and preventive approaches for early-stage COVID-19 patients could be beneficial. Meanwhile, some roles of structural and NSPs of SARS-CoV-2 in multiple complications and diseases have been discussed, suggesting that targeting these proteins could be a promising therapeutic approach have been discussed. However, more investigations are required to uncover their mechanism of action in contributing to the severity of COVID-19 and associated disease progression.

## Data Availability

Not applicable.

## Abbreviations

ALT: Alanine transaminase
Alb: Albumin
ACE2: Angiotensin-converting enzyme 2
ADCC: Antibody-dependent cell-mediated cytotoxicity
ADCP: Antibody-dependent cell-mediated phagocytosis
ADAM17: A-disintegrin and metalloprotease 17
APOA1: Apolipoprotein AI
AURKA: Aurora A kinase
AST: Aspartate transaminase
BTF3: Basic transcription factor 3
BUN: Blood urea nitrogen
CTSL: Cathepsin L1
CNS: Central nervous system; chitinase 3-like 1
C12ORF60: Chromosome 12 open reading frame 60
CHCHD2: Coiled-coil-helix-coiled-coil-helix domain containing 2
CDC: Complement-dependent cytotoxicity
COVID-19: Coronavirus disease 2019
CHI3L1: Chitinase 3-like-1
CRP: C-reactive protein
Cr: Creatinine
CLRs: C-type lectin receptors
CXCL10: C-X-C motif chemokine ligand 10
CST3: Cystatin C
DC: Dendritic cells
DMVs: Double-membrane vesicles
DYNLT1: Dynein light chain Tctex-type 1
ER: Endoplasmic reticulum
EMT: Endothelial-mesenchymal transition
Eph: Erythropoietin-producing hepatocellular
FcγRs: Fc gamma receptors
FAK: Focal adhesion kinase
Fc: Fragment
GIPC1: GAIP/RGS19-interacting protein
HNRNPD: Heterogeneous nuclear ribonucleoproteins
HE: Hemagglutinin esterase
HLA-E: Histocompatibility antigen alpha chain E
HIV: Human immunodeficiency virus
HIF-1α: Hypoxia-inducible factor α-subunits
IRF3: IFN regulatory factor 3
IKKβ: IkB kinase beta
IgG: Immunoglobulin G
IP10: Induced protein 10
IFN: Interferon
IFI35: Interferon-induced 35 kDa
IL: Interleukin
LDH: Lactate dehydrogenase
LLPS: Liquid–liquid phase separation
LDLR: Low density lipoprotein receptor
MMP: Matrix metalloproteinase
MHC: Major histocompatibility complex
M proteins: Membrane proteins
MERS: Middle East respiratory syndrome
MAVS: Mitochondrial antiviral signaling
MCP1: Monocyte chemoattractant protein-1
MHV: Mouse hepatitis virus
NRGN: Neurogranin
NRP-1: Neuropilin-1
NK: Natural killer
NSPs: Non-structural proteins
NT-PRONP: N-terminal (NT)-pro hormone BNP
N protein: Nucleocapsid protein
ORF: Open reading frame
PLT: Platelet
RdRP: RNA-dependent RNA polymerase
RBD: Receptor-binding domain
RTKs: Receptor tyrosine kinases
SPARC: Secreted protein acidic and cysteine rich
SERPINA 1: Serpin family A member 1
SARS-CoV2: Severe acute respiratory syndrome coronavirus 2
SERF: Small EDRK-rich factor 2
STAT3: Signal transducer and transcription activator 3
SIGN: Specific intercellular adhesion molecule-3-grabbing-integrin
S protein: Spike protein
TGF-β: Transforming growth factor-beta
TMPRSS2: Transmembrane protease, serine 2
TLR: Toll-like receptors
TNF-α: Tumor necrosis factor-alpha
UBL5: Ubiquitin-like protein 5
UBE2C: Ubiquitin conjugating enzyme E2 C
UTR: Untranslated region
UMOD: Uromodulin
VEGF: Vascular endothelial growth factor
